# Effect of a Substituent on the Properties of Salicylaldehyde Hydrazone Derivatives

**DOI:** 10.1021/acs.joc.2c02547

**Published:** 2023-02-03

**Authors:** Marta Hoelm, Justyna Adamczyk, Kinga Wzgarda-Raj, Marcin Palusiak

**Affiliations:** † Department of Physical Chemistry, Faculty of Chemistry, University of Lodz, Pomorska 163/165, Lodz90-236, Poland; ‡ Department of Organic and Applied Chemistry, Faculty of Chemistry, University of Lodz, Tamka 12, Lodz91-403, Poland

## Abstract

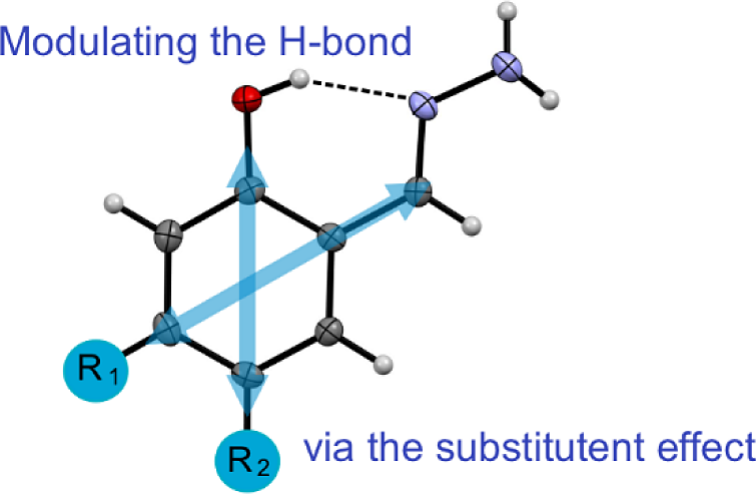

The present study investigates the effect of the substitution of salicylaldehyde hydrazones at two selected positions, i.e., the *para*-position with regard to the proton-donating and proton-accepting centers forming the hydrogen bridge. A detailed analysis of structural data obtained by theoretical approaches and X-ray experiments, together with original resonance Hammett’s constants, indicates that the strength of the intramolecular hydrogen bonding present in salicylaldehyde hydrazones can be selectively modulated by substitution of the parent molecular system with the chemical group of known π-electron-donating or -accepting properties. Our findings provide an insight into planning synthesis pathways for salicylaldehyde hydrazone species and predicting their result with regard to their H-bonding and related physical and chemical properties.

## Introduction

The most general method for synthesizing chemical species with desired properties is the substitution of a given bearing moiety with another particular functional group.^1−3^ Such substitution is a well-explored method for planned chemical synthesis and is considered one of the most basic concepts of the theory of organic and physical chemistry.^4−7^ According to IUPAC recommendations the term substituent shall be understood as “an atom or group that replaces one or more hydrogen atoms attached to a parent structure or characteristic group.”^8^ In general, the substituent effect is typically explained with regard to the properties of aromatic species^9,10^ in which the substitution of one or more hydrogen atoms, as a substituent(s), results in changes in the physical and chemical properties of the parent moiety.^11^ Furthermore, the effect can be conceptually separated into a field/inductive component (through the space/along the σ-bonds) and a resonance one (through the π-electron conjugation).^12^

The substituent effect was first quantified numerically by Hammett,^13−15^ who constructed the equation defining the substituent constants, σ, estimated as the difference between the ionization constants of substituted benzenoic acids and the ionization constant of an unsubstituted reference. Although Hammett’s equation has an empirical foundation, a theoretical methodology for substituent constant estimation has also been successfully implemented.^16^ Following Hammett’s principle, the two σ components, i.e., the field/inductive constants and the resonance constants, were numerically quantified by means of *F* and *R* parameters, respectively.^17,18^ The substituent effect has an important role in shaping various chemical and physical properties of chemical species, such as reactivity;^19−25^ however, in regard to our present work, it also influences the potential for the formation of noncovalent bonds, including hydrogen bonding^26,27^ and halogen bonding,^28^ as well as also other nonspecific interactions.^29^

The present paper studies salicylaldehyde hydrazones formed by the reaction of a modified salicylaldehyde with a primary amine, that is, hydrazine. The mechanism of this reaction is based on the nucleophilic attachment of the amine to the carbonyl group of the aldehyde and the elimination of the water molecules. Salicylaldehyde hydrazones are examples of Schiff’s bases^30^ containing an imine bond; as such, they have a range of applications in many fields of science, including the synthesis of new organic compounds. These compounds are perfect as substrates for the synthesis of nonsymmetrical salicylaldehyde azines, which are characterized by stability, intense fluorescence in the solid state, aggregation-induced emission (AIE), and exited state intramolecular proton transfer (ESIPT).^31−37^ The ESIPT compounds can present a large Stokes shift caused by the transformation of the excited enol form to the excited keto form.^38^ In general, derivatives of salicylaldehyde are known in the literature,^39,40^ but the hydrazones synthesized for the purpose of this research are characterized and reported in the form of publication for the first time.

**Scheme 1:**
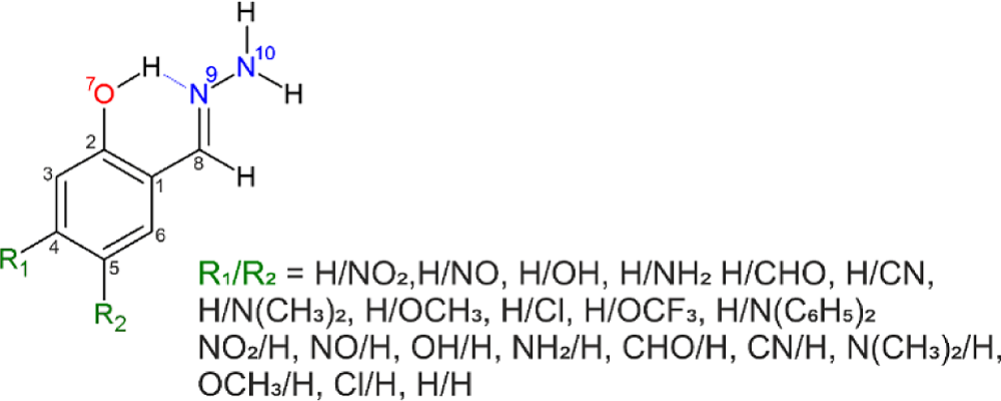
Schematic Representation of the Set of 21 Models Studied in This Work^a^ Experimental data were obtained for two of them, i.e., salicylaldehyde hydrazone substituted by −N(C_6_H_5_)_2_ (**I**) and −OCF_3_ (**II**) at position R_2_. Dotted line indicates an intramolecular hydrogen bond.

The photochromic properties of salicylaldehyde azine derivatives make them popular choices in the field of organic electronics,^41^ fluorimetric chemosensors,^42^ and fluorescent dyes.^43^ Schiff’s bases are also needed in the field of coordination chemistry, especially in complex development, as they are potentially capable of forming stable complexes with metal ions.^44^ Furthermore, compounds containing imine bonds very often show biological activity,^45^ and salicylaldehyde hydrazones are used as building blocks for structures with anticancer,^46^ antimicrobial,^47^ antifungal,^48^ and antibacterial activity.^49^ The essential structural property shared between this group of compounds is the presence of an intramolecular hydrogen bond,^50,51^ in our case formed between *ortho*-substitued hydroxyl and hydrazone group (see Scheme 1).

The intramolecular H-bond pattern present in the system is of particular interest, as it characterizes a so-called resonance-assisted hydrogen bond, first reported by Gilli and coworkers,^52−54^ and is also found in hydrazone derivatives.^55^ A thorough introduction to the RAHB topic is given by Domagała et al.^56^ Because the extra-ring formed via the hydrogen bonding is a π-electron conjugated along the sequence of formally covalent bonds, it may be also classified as a quasi-aromatic ring.^57^ Both aromatic and quasi-aromatic rings are fused in this case and strongly interact with themselves, as previously observed in structurally related systems.^58−60^ The interaction between the two rings happens mostly via the resonance effect, and as such, it may be effectively modulated by the substitution effect.^61−64^ This paper examines the influence of the substitution of the benzene ring in positions 4 and 5 on the varied properties of salicylaldehyde hydrazones.

A theoretical review of the substituent effect is followed by a discussion of the results of the synthesis of two salicylaldehyde hydrazone derivatives, including a structural analysis based on a single-crystal X-ray examination. These two real systems are then discussed against the background of a theoretical investigation of a set of 21 representative models modified by substituents covering the largest possible spectrum of resonance effect abilities.

## Experimental Section

### Quantum-Chemical Calculations

The energetic and molecular properties of all structures (Scheme 1), including both close (with IMHB) and open (without IMHB) tautomeric forms (a total of 21 systems in two tautomeric forms) were determined using the density functional theory method (DFT).

Calculations were performed in the gas phase at the B3LYP-GD3/aug-cc-pVTZ level of theory in the Gaussian 09 program (Revision D.01).^65^ B3LYP was chosen because it is the most widely used hybrid functional for determining the equilibrium geometries of many compounds, including hydrogen-bonded systems,^66−69^ while the Dunning’s correlation-consistent polarized basis set aug-cc-pVTZ gives accurate results with considerable efficiency.^70^ In addition, ultrafine integration grids were utilized to increase the accuracy of the numerical integration during optimizations. The harmonic vibrational calculations were performed at the same theory level as optimization, and the absence of imaginary frequencies confirms that the obtained structures are local minima on the potential energy surface.

The energetical parameter Δ_*E*_c/o_ was calculated as a difference between the energy of closed (with HB) and open (no HB) optimized tautomers shown in Scheme 1.

The aromaticity of the quasi-aromatic ring was investigated by means of the geometry-based aromaticity indicator HOMA (harmonic oscillator model of aromaticity). It was developed by Krygowski and Kruszewski^71,72^ and is expressed as follows:
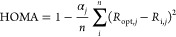
1where: α_*j*_ is an empirical constant, *j* denotes the type of bond, *n* is the number of bonds taken into the summation, and *R*_opt_ and *R*_i_ are optimal and individual bond lengths, respectively.

Only the C–O, C–C, and C–N bonds were considered to estimate the π-electron delocalization s = degree in the quasi-ring. According to recent HOMA index actualization, the C–O and C=O bonds: α = 157.38 and *R*_opt_ = 1.265 Å, for the C–C bond: α = 257.7 and *R*_opt_ = 1.388 Å, while for the C=N bond: α = 93.52 and *R*_opt_ = 1.334 Å.^73^

### Synthesis and X-ray Experiment

Compounds (**I**) and (**II**) were obtained along the earlier reported method of synthesis^74^ as pale yellow solids. Single crystals suitable for X-ray analysis were obtained by slow evaporation of the solution on (**I**) and (**II**) in a mixture of hexane/dichloromethane (5:5). See the Supporting Information for details of the synthesis and all products’ full spectral characterization (^1^H-NMR, ^13^C-NMR, IR).

X-ray diffraction data for (**I**) and (**II**) were collected on an XtaLAB Synergy, Dualflex, HyPix diffractometer. Integration of the intensities and corrections for Lorentz effects, polarization effects, and analytical absorption were performed with CrysAlis PRO.^75^ Using Olex2,^76^ the structure was solved with the SHELXT^77^ structure solution program using Intrinsic Phasing and refined with the SHELXL^78^ refinement package using Least Squares minimization. The hydrogen atoms of aromatic rings were introduced in the calculated positions with an idealized geometry and constrained using a rigid body model with isotropic displacement parameters equal to 1.2 of the equivalent displacement parameters of their parent atoms. The positions of the hydrogen atoms of the −NH_2_ groups were found on a Fourier difference map and refined isotropically without any restraints. The molecular geometries were calculated by the PLATON program.^79^ The relevant crystallographic data are given in Table S1 (SI). Atomic coordinates, displacement parameters, and structural factors of the analyzed crystal structures are deposited with the Cambridge Crystallographic Data Centre CCDC (reference number: 2214343 for (**I**) and 2214344 for (**II**)).^80^

## Results and Discussion

### Substituent Effect on Salicylaldehyde Hydrazones

The study examined the effects of substitution on the geometrical and electronic properties of salicylaldehyde hydrazone derivatives. For this purpose, various electron-donating (EDG) and electron-withdrawing (EWG) groups (substituents) were chosen and attached to benzene at the R_1_ and R_2_ positions (Scheme 1). The geometries of all structures obtained from the B3LYP-GD3 optimizations are shown in Table S6 (SI). In these molecules, an intramolecular hydrogen bond (IMHB) is formed between hydrazone (−NNH_2_) and hydroxyl (−OH) groups (Scheme 1). The geometrical parameters describing this interaction, as well as the N9–N10 bond lengths are summarized in Table 1 together with Hammett’s resonance substituent constants (*R*).^12,13,81^

**Table 1:** Geometrical Parameters of the Hydrogen Bonds, Together with N–N Bond Length, Obtained from the B3LYP-GD3/aug-cc-pVTZ Calculations^a^

molecule		geometrical parameters of IMHB					
R_1_	R_2_	*d*_O7–H7_ [Å]	*d*_H7···N9_ [Å]	*d*_O7···N9_ [Å]	<O7–H7···N9 [°]	*d*_N9–N10_ [Å]	R
H	CHO	0.986	1.775	2.650	145.9	1.369	0.0900
	Cl	0.983	1.796	2.664	145.3	1.370	–0.1900
	CN	0.985	1.780	2.652	145.6	1.367	0.1500
	N(CH_3_)_2_	0.980	1.816	2.678	144.9	1.376	–0.9800
	NH_2_	0.980	1.813	2.676	145.0	1.375	–0.7400
	NO	0.987	1.766	2.643	146.1	1.367	0.4200
	NO_2_	0.987	1.773	2.648	145.8	1.366	0.1300
	OCH_3_	0.981	1.806	2.670	145.1	1.375	–0.5600
	OH	0.981	1.809	2.673	145.0	1.373	–0.7000
	N(C_6_H_5_)_2_ (I)	0.982	1.800	2.667	145.3	1.373	–0.3400
	OCF_3_ (II)	0.983	1.793	2.661	145.4	1.369	–0.0400
	H	0.983	1.795	2.664	145.5	1.374	0.0000
CHO	H	0.983	1.796	2.662	145.2	1.366	0.0900
Cl		0.984	1.788	2.659	145.6	1.372	–0.1900
CN		0.983	1.791	2.659	145.3	1.365	0.1500
N(CH_3_)_2_		0.984	1.790	2.665	146.2	1.384	–0.9800
NH_2_		0.985	1.785	2.661	146.2	1.382	–0.7400
NO		0.982	1.797	2.662	145.0	1.362	0.4200
NO_2_		0.983	1.794	2.660	145.1	1.362	0.1300
OCH_3_		0.984	1.791	2.664	146.0	1.379	–0.5600
OH		0.985	1.786	2.660	146.0	1.379	–0.7000

a The last column contains Hammett’s resonance substituent constants (*R*).^12,13,81^ The atom numbers are shown in Scheme 1.

In keeping with the topic of this work, this section will first discuss the influence of substituent type (EDG vs EWG) on the strength of the IMHB under investigation. The HB under discussion is defined by the structural parameter *d*_H···N_ distance: this is a widely known and thoroughly accepted structural measure of H-bonding strength which is well correlated with other structural parameters and energy parameters, and can be easily applied in further studies using experimental X-ray data.^82^ It should be noted that while other similar parameters such as the *d*_O···N_ distance (also collected in Table 1) could also be used, *d*_H···N_ uses a wider range of values and is hence more sensitive to changes due to the substituent effect. Nevertheless, the correlation coefficient *R*_c-c_ for the linear relationship between the two parameters is about 0.97.

From the first observation, it can be concluded that the substitution at the R_2_ position results in significantly larger differences in H-bond parameters than at the R_1_ position. For instance, the span of *d*_O···H_ values is 0.050 Å for R_2_ but only 0.012 Å for R_1_. In other words, it can be said that the influence of substituents at the R_2_ position is about five times as influential as in the R_1_ position. This is an important consideration when planning the chemical synthesis of related chemical species.

**Figure 1:**
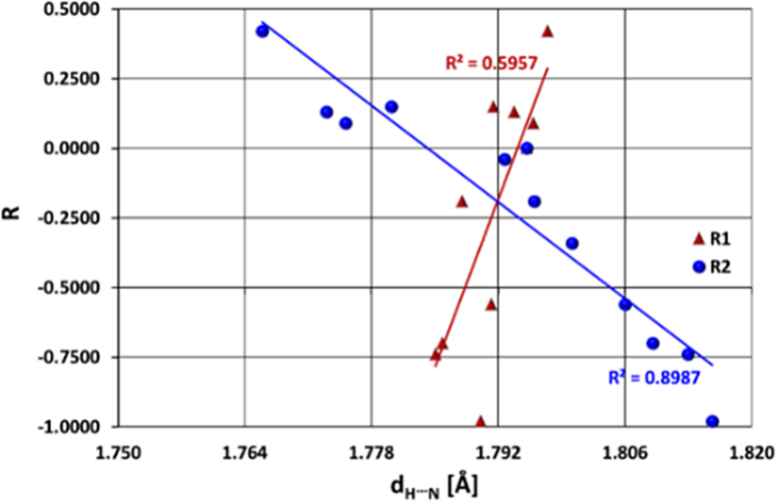
Relationship between *d*_H···N_ distance (in Å) and Hammett’s resonance constant (*R*) for the investigated cases. Circles and triangles correspond to systems substituted at the R_2_ and R_1_ positions, respectively.

Another significant finding arises when comparing the H···N distance in the H-bridge (the most commonly used structural indicator of HB strength)^82^ with the Hammett resonance effect constant^12^ (Table 1). It appears that while a straight linear correlation can be seen between *d*_H···N_ and the R constant in the case of substitution at position R_2_, no such interrelation is observed for systems substituted at position R_1_ (Figure 1). This implies that position R_2_ would be a more favorable target for further synthesis, in which a greater influence on H-bonding strength is desired.

**Scheme 2:**
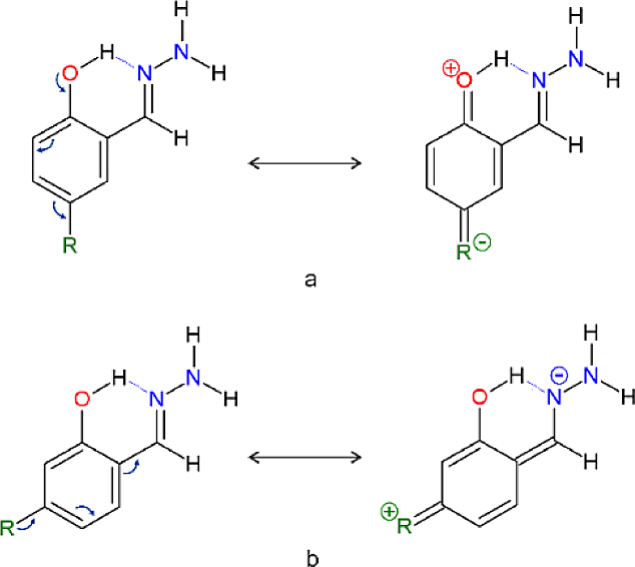
Charge-Separated Resonance Structure Contributions which Enhance H-Bonding

Based on Figure 1, it can be said that, generally, more electron-withdrawing substituents at position R_2_ are associated with stronger H-bonds, i.e., a shorter *d*_H···N_ distance. The opposite general trend can be observed for R_1_ substitution; however, as mentioned earlier, the influence of substituents in this case is less efficient and not as regular due to a lack of linear relationships. This observation can be accounted for by the contribution of the given charge-separated resonance structures (Scheme 2) and the fact that both effects (a) and (b), shown in the scheme, favor H-bond formation. Hence, it would be reasonable to expect that EDG in position R_2_ and EWG in position R_1_ will weaken the H-bonding; in fact, this is the case, since longer *d*_H···N_ distances are observed for substituents with greater electron-donating properties.

**Figure 2:**
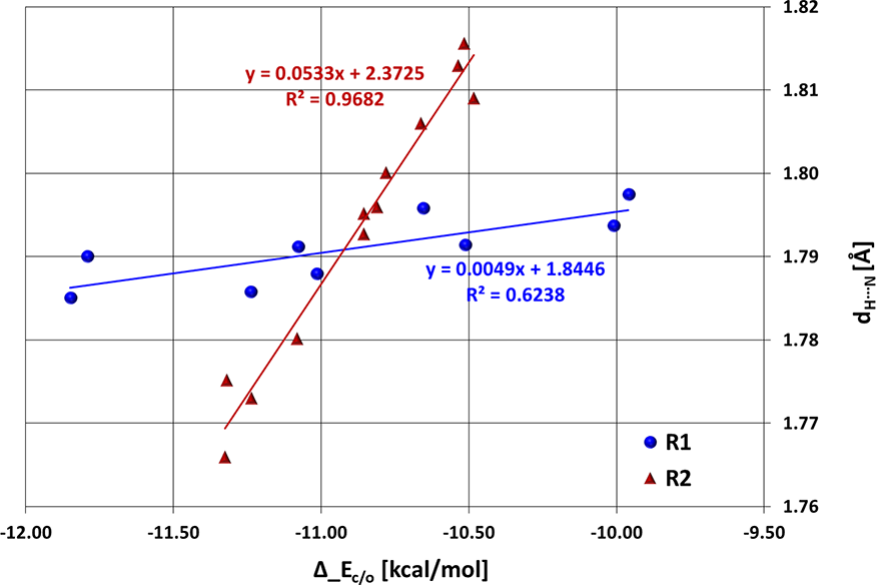
Linear relation between HB distance and HB/nonHB tautomer energy difference (Δ_*E*_c/o_) separated into two trends, for R_1_ and R_2_ substitution, respectively.

To have an inside into the energy parameters of the HB under investigation, we have estimated a difference between closed (with HB) and open (no HB) tautomers of structures from Scheme 1. Although such energy difference is not an HB energy itself (for intramolecular interactions such energy cannot be defined explicitly), this parameter can be effectively used as a measure of H-bonding strength.^83^ It appears that for all cases the Δ_*E*_c/o_ follows the *d*_H···N_ distance, as it should be expected.^82^ The span of values is in the range of 9.96–11.85 kcal/mol, while the difference between the maximum and minimum values is 1.89 kcal/mol—this corresponds to about 17% of the energy estimation of the HB strength. For comparison, the exact HB energy in the water dimer is about 5 kcal/mol.^84^ Interestingly, data can be separated into two independent trends, for R_1_ and R_2_ substitution, respectively. See Figure 2 for a graphical interpretation. For R_2_ substitution, the trend is clearly linear (*R* coefficient close to 1), while for R_1_, the linear relation can hardly be noticed (*R* coefficient about 0.6). Additionally, the slope of the linear function indicates the R_2_ substitution as much more effective (the slope parameter is about 10 times greater for R_2_ substitution with respect to R_1_ substitution). It is also worth pointing out that in both R_1_ and R_2_ substitutions, there is a close to linear trend of changes between the Δ_*E*_c/o_ and the Hammett constant (correlation coefficients *R* about 0.9); importantly, the slopes of linear approximations are opposite, which confirms our earlier conclusion (more electron-withdrawing substituents at position R_2_ are associated with stronger H-bonds and opposite for R_1_). See Figure S9 for a graphical illustration.

**Scheme 3:**
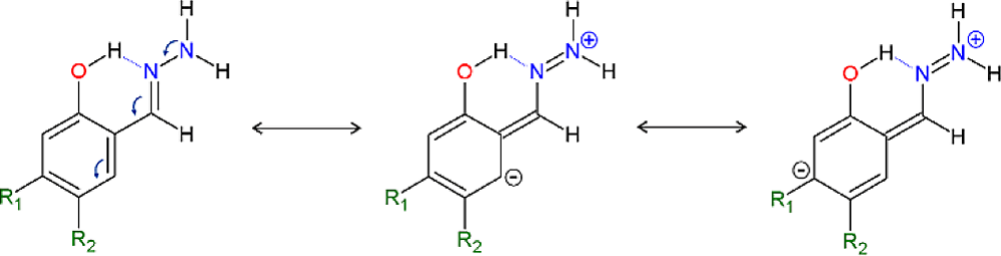
Resonance Structures Illustrating the Interaction between the Hydrazine Group and the Phenyl Moiety

This begs the question of why R_2_ substitution is more effective than R_1_ substitution, insofar that it appears to have a greater influence on the HB. The answer may lie in the properties of the −NH_2_ group in the hydrazine-containing −C(*H*)N-NH_2_ moiety. The −C(*H*)N-NH_2_ group possesses its own resonance effect in which the −NH_2_ acts as an electron-donating fragment, transferring a formal negative charge to the adjacent N atom. This situation is presented as formal charge separation resonance structures in Scheme 3. As a consequence, the formal charge surplus is transferred to the phenyl ring, into the *ortho*- and *para*-positions with respect to the −CNNH_2_ group, potentially also involving the R_1_-attached substituents. Obviously, this resonance path is; (i) favored for electron-withdrawing substituents at the R_1_ position; (ii) competitive for the resonance paths illustrated in Scheme 2 and, finally, (iii) it has less influence on the H-bond itself, and for this reason, the substitution in position R_1_ generally has less influence on the HB under investigation.

**Figure 3:**
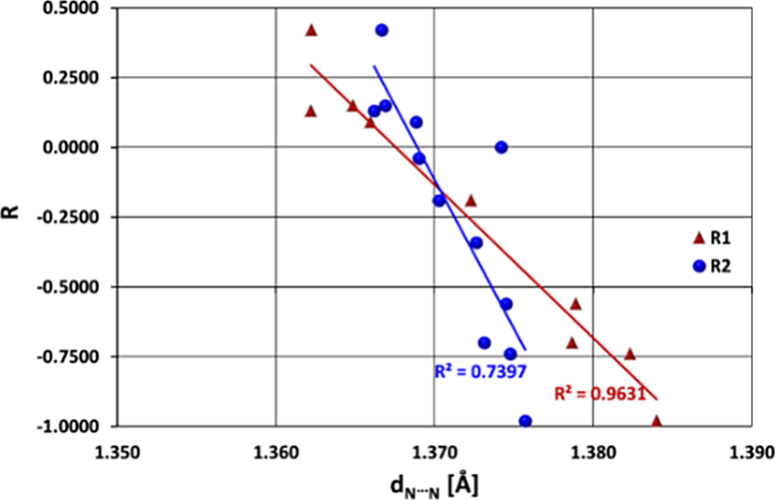
Correlation between *d*_N–N_ (in Å) and substituent constants (*R*) for systems substituted in the R_1_ and R_2_ positions.

As a consequence of the above interrelations, a good linear relationship (correlation coefficient *R*_C-C_ = 0.98, see Figure 3) exists between the N–N distance (*d*_N–N_) and the substituting constants for R_1_ substituted systems: the N–N bond lengthens with greater electron-donating properties of the substituent at R_1_. Conversely, this relationship is less favorable for R_2_-substitution: the N–N bond is longer when the groups at R_2_ are more electron-donating (correlation coefficient *R*_c-c_ = 0.86).

Another key observation can be made at this point. The substitution at position R_2_ appears to be “aimed” at the hydroxyl group, the proton-donating group in the H-bridge, while the substitution at position R_1_ is “aimed” toward the −C(*H*)N-NH_2_ group, in which the inner nitrogen atom acts as a proton-accepting center. Our results suggest that of the two configurations, it is easier to affect the −OH group, since it has no resonance effect of its own, and the proton-donating center is directly attached to the benzene ring: the transferring moiety for the substituent effect.

**Figure 4:**
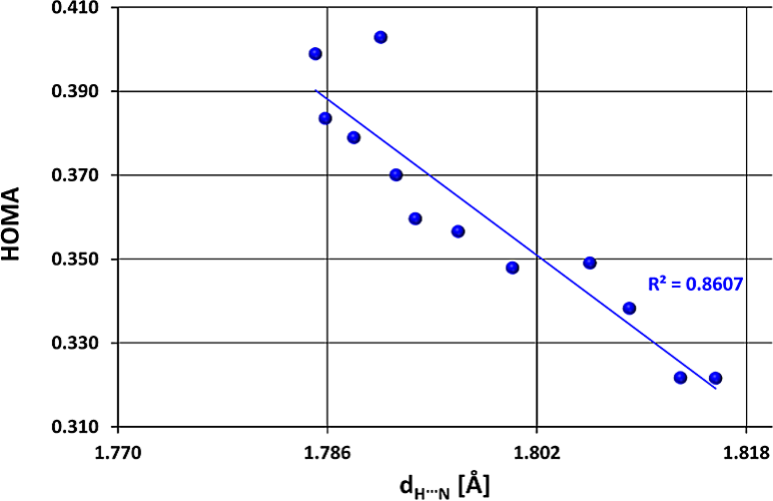
π-Electron delocalization in the quasi-ring, expressed by HOMA values, as a function of *d*_H···N_ (in Å) in H-bridge.

The IMHB investigated in this work may be classified as a typical resonance-assisted hydrogen bond.^54,56^ As such, it should be characterized by an observable relationship between the degree of π-electron delocalization within the quasi-ring and the strength of the HB, here reflected by its structural properties (*d*_H···N_ length). This relationship can only be observed for electron-donating substituents; however, this includes all representatives in this case, including both R_1_ and R_2_ substitutions (Figure 4).

Interestingly, in the case of EWG, no such relationship can be observed in either the R_1_ or R_2_ position, only a chaotic distribution of points and a much lower span of HOMA (Table S7 in SI). That may be due to the fact that in the case of EWGs, the resonance effect to which they contribute (Scheme 2), and the inner quasi-ring resonance effect, supported by −C(*H*)N-NH_2_, is competitive in nature.

### X-ray Structural Studies of the Solid State

How do these conclusions relate to the two experimentally investigated systems, i.e., salicylaldehyde hydrazone substituted by −N(C_6_H_5_)_2_ (compound **I**) and −OCF_3_ (compound **II**) at position R_2_? Only these two compounds were available, as although a number of chemical derivatives were tested, i.e., those substituted by OCH_3_/H, H/F, H/C_6_H_5_, H/NO_2_, H/C_6_H_5_(4-N(C_6_H_5_)_2_), H/C_6_H_5_(4-CF_3_) in position R_1_/R_2_, it was only possible to obtain single-crystal samples suitable for X-ray diffraction for (**I**) and (**II**). All compounds were obtained according to the known method of condensation synthesis of an aldehyde molecule with hydrazine hydrate.^74^ Hence, our present discussion must be limited to the structural properties of IMHB present in these two obtained crystal structures. A detailed description of both structures, including the intermolecular interactions and packing, can be found in the SI associated with this manuscript.

**Figure 5:**
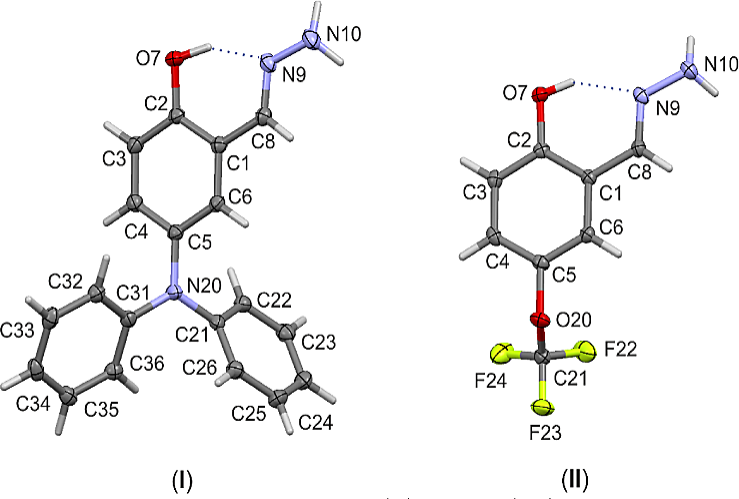
Molecular structure of (**I**) and (**II**) with atom labeling scheme. Displacement ellipsoids are drawn at a 50% probability level. The dotted line indicates an intramolecular hydrogen bond.

In both crystal structures (**I**) and (**II**), the presence of hydrazone- and hydroxy-groups leads to the formation of the expected IMHB, geometrical parameters are collected in Table 2. As seen in Figure 5, an intramolecular O7–H7···N9 hydrogen bond is formed between the −OH group and the N atom from the −CNNH_2_ group. This results in the formation of a cyclic S(6) motif, according to the graph-set method of Abs classification.^85^ The differences in the distances in the H-bridges between the two structures may seem relatively small at the first sight; however, they appear considerably larger when comparing them with theoretical calculations (Table 1). Regardless of the structural parameter used for assessment, the H-bond in structure (**I**), that is, substituted by the −N(C_6_H_5_)_2_ group at position R_2_, appears slightly stronger than its counterpart in structure (**II**). This observation is perfectly in line with the results of our theoretical calculations. Both substituents in the experimentally investigated structures demonstrate electron-donating properties via the resonance effect; in contrast, the electron-withdrawing structures (Scheme 2a) should weaken the H-bonding along with their electron-donating properties. The −N(C_6_H_5_)_2_ group has stronger electron-donating properties than the -OCF_3_ group, as expressed by an R substituent constant of −0.34 for the −N(C_6_H_5_)_2_ group compared to −0.04 for the −OCF_3_ group. As a consequence, the *d*_H···N_ and *d*_O···N_ contacts in the H-bridge appear longer in (**I**) than in (**II**). The effect of substitution on H-bonding is observable and follows our theoretical analysis.

**Table 2:** Geometric Parameters of Intramolecular Hydrogen Bonds – Distances [Å] and Angles [°]

IMHB	D–H	H···A	D···A	D–H···A
(I)				
O(7)–H(7)···N(9)	0.840	1.895	2.6340(12)	146.12
(II)				
O(7)–H(7)···N(9)	0.840	1.889	2.6298(13)	146.39

## Conclusions

Based on our theoretical and experimental analysis, the R_2_ position of salicylaldehyde hydrazones appears a more attractive target for organic synthesis in cases where it is intended to use the substituent effect to influence the H-bonding and its all assisting effects, including π-electron conjugation in the extra quasi-ring. The presence of electron-withdrawing groups in this position favors a stronger hydrogen bond while electron-donating groups weaken this interaction. Substitution in position R_1_ is about five times less effective compared to position R_2_. The same can be concluded when comparing the energy difference of the closed and open tautomers of the investigated molecular system.

Additionally, the character of the substituents (electron-withdrawing vs electron-donating via the resonance effect) has the opposite influence on hydrogen bonding in the R_2_ position, that is, the electron-donating ones favor H-bonding. These changes in hydrogen bonding parameters can be explained via resonance charge-separated structures and are strictly correlated with Hammett’s resonance substituent constants. The results of our analysis of two representative crystal structures, obtained by X-ray single crystallography, confirm the results of theoretical calculations performed for a set of 21 representative systems from the same group with various other substitutions.

## Data Availability

The data underlying this study are available in the published article and its supporting information.

## Supporting Information Available

Materials, Synthesis and Spectroscopic methods for 2-hydroxy-5-(*N*-phenylanilino)benzaldehyde, compounds (I) and (II); Crystallographic research for compounds (I) and (II); ^1^H-NMR for 2-hydroxy-5-(*N*-phenylanilino)benzaldehyde (Figure S1); ^1^H-NMR and ^13^C-NMR for compound(I) (Figure S2); ^1^H-NMR and ^13^C-NMR for compound (II) (Figure S3); IR for compound (I) (Figure S4); IR for compound(II) (Figure S5); Details of the crystal structure determination of (I) and (II) (Table S1); Molecular structure of (I) and (II) with atom labeling scheme (Figure S6); Geometric parameters for crystal structure (I) (Table S2); Geometric parameters for crystal structure (II) (Table S3); Scheme of hydrogen bonds presented in the crystal structures (I) and (II) (Figure S7); Geometric parameters of selected hydrogen bonds for crystal structures (I) and (II) (Table S4); View of the chain of molecules by (II) – (II) intermolecular interactions and view of the unit cell showing the herringbone arrangement of molecules (Figure S8); Total energies as well as the energy differences (Δ_*E*_c/o_) calculated between closed and open tautomeric forms of the analyzed molecules obtained from the B3LYP-GD3/aug-cc-pVTZ calculations (Table S5); The correlation between energy difference (Δ_*E*_c/o_) and the Hammett’s constant (*R*) for the investigated systems (Figure S9); Graphical representation and coordinates of the analyzed molecules obtained from the B3LYP-GD3/aug-cc-pVTZ calculations (Table S6); Values of HOMA index calculated for benzene and the quasi-ring (Table S7) (PDF)

## References

[ref1] Balaban A. T.; Schleyer P. V. R.; Rzepa H. S. Not Armit and Robinson, Begat the Six Aromatic Electrons. Chem. Rev. 2005, 105, 3436–3447. 10.1021/cr0300946. 16218557

[ref2] Chen Z.; Wannere C. S.; Corminboeuf C.; Puchta R.; Schleyer P. V. R. Nucleus-independent chemical shifts (NICS) as an aromaticity criterion. Chem. Rev. 2005, 105, 3842–3888. 10.1021/cr030088+. 16218569

[ref3] Vilkov I. V.; Mastryukov V. S.; Sadova N. I. Foreword by Roald Hoffmann to: Determination of the Geometrical Structure of Free Molecules; Mir Publishers: Moscow, 1983.

[ref4] Exner O.; Krygowski T. The nitro group as substituent. Chem. Soc. Rev. 1996, 25, 71–75. 10.1039/cs9962500071.

[ref5] Krygowski T. M.; Stepien B. T. Sigma and Pi-Electron Delocalization: Focus on Substituted Effects. Chem. Rev. 2005, 105, 3482–3512. 10.1021/cr030081s. 16218559

[ref6] Exner O.; Böhm S. Theory of Substituent Effects: Recent Advances. Curr. Org. Chem. 2006, 10, 763–778. 10.2174/138527206776818892.

[ref7] Sykes P. A guidebook to mechanism in organic chemistry, 6th ed.; Pearson Education Limited: Harlow, England, 1986.

[ref8] IUPAC. Compendium of Chemical Terminology, 2nd ed. (the ″Gold Book″) compiled by A. D. McNaught and A. Wilkinson; Blackwell Scientific Publications: Oxford, 1997. XML on-line corrected version: http://goldbook.iupac.org created by Nic, M.; Jirat, J.; Kosata B.; updates compiled by Jenkins, A., **2006**.

[ref9] Lloyd D. The chemistry of conjugated cyclic compounds: to be or not to be like benzene; Wiley: New York, 1990.

[ref10] Smith M. B.; March J. March’s Advanced Organic Chemistry: Reactions, Mechanisms and Structure; Wiley: New York, 5th ed., 2001.

[ref11] Muller P. Glossary of terms used in physical organic chemistry. Pure Appl. Chem. 1994, 66, 1077–1184. 10.1351/pac199466051077.

[ref12] Hansch C.; Leo A.; Taft R. W. A survey of Hammett substituent constants and resonance and field parameters. Chem. Rev. 1991, 91, 165–195. 10.1021/cr00002a004.

[ref13] Hammett L. P. The effect of structure upon the reactions of organic compounds Benzene derivatives. J. Am. Chem. Soc. 1937, 59, 96–103. 10.1021/ja01280a022.

[ref14] Leffler J.E.; Grunwald E. Rates and Equilibria of Organic Reactions; John Wiley: New York, 1963.

[ref15] Chapman N. B.; Shorter J. Correlation Analysis in Chemistry; Plenum Press: New York, 1978.

[ref16] Jezuita A.; Ejsmont K.; Szatylowicz H. Substituent effects of nitro group in cyclic compounds. Struct. Chem. 2021, 32, 179–203. 10.1007/s11224-020-01612-x.

[ref17] Swain C. G.; Lupton E. C. Field and resonance components of substituent effects. J. Am. Chem. Soc. 1968, 90, 4328–4337. 10.1021/ja01018a024.

[ref18] Taft R. W.; Lewis I. C. Evaluation of Resonance Effects on Reactivity by Application of the Linear Inductive Energy Relationship V. Concerning a σR Scale of Resonance. J. Am. Chem. Soc. 1959, 81, 5343–5352. 10.1021/ja01529a025.

[ref19] Sykes P. A guidebook to mechanism in organic chemistry, 6th ed.; Pearson Education Limited: Harlow, England, 1986; p 416.

[ref20] Smith M. B.; March J. March’s Advanced Organic Chemistry: Reactions, Mechanisms, and Structure; Wiley: NJ, 2007.

[ref21] Palusiak M.; Domagała M.; Dominikowska J.; Bickelhaupt F. M. The substituent effect on benzene dications. Phys. Chem. Chem. Phys. 2014, 16, 4752–4763. 10.1039/C3CP54089H. 24469543

[ref22] Matta Ch F. Modeling biophysical and biological properties from the characteristics of the molecular electron density, electron localization and delocalization matrices, and the electrostatic potential. J. Comput. Chem. 2014, 35, 1165–1198. 10.1002/jcc.23608. 24777743 PMC4368384

[ref23] Shahamirian M.; Szatylowicz H.; Krygowski T. M. How OH and O– groups affect electronic structure of meta-substituted and para-substituted phenols and phenolates. Struct. Chem. 2017, 28, 1563–1572. 10.1007/s11224-017-0965-4.

[ref24] Szatylowicz H.; Domanski M. A.; Krygowski T. M. Classical and Reverse Substituent Effects in Substituted Anthrol Derivatives. Open Chem. 2019, 8, 64–73. 10.1002/open.201800234. PMC6346296 30697512

[ref25] Remya G. S.; Suresh Ch H. Quantification and classification of substituent effects in organic chemistry: a theoretical molecular electrostatic potential study. Phys. Chem. Chem. Phys. 2016, 18, 20615–20626. 10.1039/C6CP02936A. 27412764

[ref26] Szatylowicz H.; Krygowski T. M. Effect of the Substituent and Hydrogen Bond on the Geometry and Electronic Properties of OH and O– Groups in para-Substituted Phenol and Phenolate Derivatives. J. Phys. Chem. A 2010, 114, 10885–10890. 10.1021/jp1071204. 20853885

[ref27] Wieczorkiewicz P. A.; Szatylowicz H.; Krygowski T. M. Mutual relations between substituent effect, hydrogen bonding, and aromaticity in adenine-uracil and adenine-adenine base pairs. Molecules 2020, 25, 3688 10.3390/molecules25163688. 32823565 PMC7464026

[ref28] Domagała M.; Palusiak M. The influence of substituent effect on noncovalent interactions in ternary complexes stabilized by hydrogen-bonding and halogen-bonding. Comput. Theor. Chem. 2014, 1027, 173–178. 10.1016/j.comptc.2013.11.007.

[ref29] Lewis M.; Bagwill C.; Hardebeck L. K. E.; Wireduaah S. The use of Hammett constants to understand the non-covalent binding of aromatics. Comput. Struct. Biotechnol. J. 2012, 1, e201204004 10.5936/csbj.201204004. 24688634 PMC3962106

[ref30] Schiff H. Mittheilungen aus dem Universitätslaboratorium in Pisa: Eine neue Reihe organischer Basen. Justus Liebigs Ann. Chem. 1864, 131, 118–119. 10.1002/jlac.18641310113.

[ref31] Kagatikar S.; Sunil D. Aggregation-induced emission of azines: An up-to-date review. J. Mol. Liq. 2019, 92, e111371 10.1016/j.molliq.2019.111371.

[ref32] Wang Z.; Zhou F.; Wang J.; Zhao Z.; Qin A.; Yu Z.; Tang B. Z. Electronic effect on the optical properties and sensing ability of AIEgens with ESIPT process based on salicylaldehyde azine. Sci. China: Chem. 2018, 61, 76–87. 10.1007/s11426-017-9147-0.

[ref33] Devi M.; Sharma P.; Kumar A.; Kaur S.; Kumar M.; Bhalla V. ESIPT Active Assemblies for ‘On-On’ Detection, Cell Imaging and Hampering Cellular Activity of 2,6-Dichloro-4-nitroaniline. Chem. – Asian J. 2022, 17, e202101219 10.1002/asia.202101219. 34942037

[ref34] Wang C.; Liu Z. Y.; Huang C.; Chen C.; Meng F. Y.; Liao Y.; Liu Y. H.; Chang C.; Li E. Y.; Chou P. T. Chapter Open for the Excited-State Intramolecular Thiol Proton Transfer in the Room-Temperature Solution. J. Am. Chem. Soc. 2021, 143, 12715–12724. 10.1021/jacs.1c05602. 34355563

[ref35] Liu X.; Liu X.; Shen Y.; Gu B. A Simple Water-Soluble ESIPT Fluorescent Probe for Fluoride Ion with Large Stokes Shift in Living Cells. ACS Omega 2020, 5, 21684–21688. 10.1021/acsomega.0c02589. 32905448 PMC7469414

[ref36] Liu Z. Y.; Hu J. W.; Huang T. H.; Chen K. Y.; Chou P. T. Excited-state intramolecular proton transfer in the kinetic-control regime. Phys. Chem. Chem. Phys. 2020, 22, 22271–22278. 10.1039/D0CP03408H. 33001109

[ref37] Jacquemin D.; Khelladi M.; Nicola A.; Ulrich G. Turning ESIPT-Based triazine fluorophores into dual emitters: From theory to experiment. Dyes Pigm. 2019, 163, 475–482. 10.1016/j.dyepig.2018.12.023.

[ref38] Zhang H.; Liu S.; Zhang C.; Fan J.; Lin L.; Wang C.; Song Y. The mechanism of the excited-state proton transfer of Salicylaldehyde azine and 2,2′-[1,4-Phenylenebis{(E)- nitrilomethylidyne}] bisphenol: Via single or double proton transfer. Spectrochim. Acta, Part A 2019, 223, e117321 10.1016/j.saa.2019.117321. 31277029

[ref39] Fei-Hua L. Theoretical study of the Substituent Effect on the Electronic Structure and Spectral Properties of Six Salicylaldehyde Schiff Bases. Russ. J. Phys. Chem. 2020, 94, 352–359. 10.1134/S0036024420020235.

[ref40] Mijanuddin M.; Sheldrick W. S.; Mayer-Figge H.; Ali M.; Chattopadhyay N. Crystal structure and feasibility of intramolecular proton transfer reaction of salicylaldazine. J. Mol. Struct. 2004, 693, 161–165. 10.1016/j.molstruc.2004.02.030.

[ref41] Adamczyk J. A.; Zielonka K.; Kotarba S.; Saramak J.; Glowacki I.; Rachwalski M.; Pieczonka A. M. Photophysical properties of novel fluorescent thin solid layers based on the Aggregation Induced Emission of alkoxy-substituted salicylaldehyde azines. J. Lumin. 2021, 229, 117668 10.1016/j.jlumin.2020.117668.

[ref42] Pei P. X.; Hu J. H.; Long C.; Ni P. W. A novel colorimetric and “turn-on” fluorimetric chemosensor for selective recognition of CN– ions based on asymmetric azine derivatives in aqueous media. Spectrochim. Acta, Part A 2018, 198, 182–187. 10.1016/j.saa.2018.03.022. 29547819

[ref43] Shen S.; Liu X.; Sunc J.; Wang M.; Jiang Z.; Xia G.; Wang H. Excited state intramolecular single proton transfer mechanism of pigment yellow 101 in solid state: Experiment and DFT calculation. Spectrochim. Acta, Part A 2019, 217, 93–100. 10.1016/j.saa.2019.03.076. 30928839

[ref44] Kumar S.; Dhar D. N.; Saxena P. N. Applications of Metal Complexes of Schiff Bases—A Review. J. Sci. Ind. Res. 2009, 2009, 181–187.

[ref45] Ganga M.; Sankaran K. R. Synthesis, spectral characterization, DFT, docking studies and cytotoxic evaluation of 1-(4-fluorobenzyl)-2,4,5-triphenyl-1H-imidazole derivatives. Chem. Data Collect. 2020, 28, 100412 10.1016/j.cdc.2020.100412.

[ref46] Gupta S. D.; Revathi B.; Mazaira G. I.; Galigniana M. D.; Subrahmanyam C. V. S.; Gowrishankar N. L.; Raghavendra N. M. 2,4-dihydroxy benzaldehyde derived Schiff bases as small molecule Hsp90 inhibitors: Rational identification of a new anticancer lead. Bioorg. Chem. 2015, 59, 97–105. 10.1016/j.bioorg.2015.02.003. 25727264

[ref47] da Silva C. M.; da Silva D. L.; Modolo L. V.; Alves R. B.; de Resende M. A.; Martins C. V. B.; de Fatima A. Schiff Bases: A Short Review of Their Antimicrobial Activities. J. Adv. Res. 2011, 2, 1–8. 10.1016/j.jare.2010.05.004.

[ref48] Backes G. L.; Neumann D. M.; Jursic B. S. Synthesis and antifungal activity of substituted salicylaldehyde hydrazones, hydrazides and sulfohydrazides. Bioorg. Med. Chem. 2014, 22, 4629–4636. 10.1016/j.bmc.2014.07.022. 25127462

[ref49] Chiter C.; Bouchama A.; Mouas T. N.; Allal H.; Yahiaoui M.; Warad I.; Zarrouk A.; Djedouani A. Synthesis, crystal structure, spectroscopic and hirshfeld surface analysis, NCI-RDG DFT computations and antibacterial activity of new asymmetrical azines. J. Mol. Struct. 2020, 1217, e128376 10.1016/j.molstruc.2020.128376.

[ref50] Kwocz A.; Kochel A.; Chudoba D.; Filarowski A. Tautomeric design of ortho-hydroxyheterocyclic Schiff bases. J. Mol. Struct. 2015, 1080, 52–56. 10.1016/j.molstruc.2014.09.073.

[ref51] Panek J. J.; Filarowski A.; Jezierska-Mazzarello A. Impact of proton transfer phenomena on the electronic structure of model Schiff bases: An AIM/NBO/ELF study. J. Chem. Phys. 2013, 139, 154312 10.1063/1.4825098. 24160518

[ref52] Gilli G.; Bellucci F.; Ferretti V.; Bertolasi V. Evidence for resonance-assisted hydrogen bonding from crystal-structure correlations on the enol form of the β-diketone fragment. J. Am. Chem. Soc. 1989, 111, 1023–1028. 10.1021/ja00185a035.

[ref53] Bertolasi V.; Gilli P.; Ferretti V.; Gilli G. Evidence for resonance-assisted hydrogen bonding. 2. Intercorrelation between crystal structure and spectroscopic parameters in eight intramolecularly hydrogen bonded 1,3-diaryl-1,3-propanedione enols. J. Am. Chem. Soc. 1991, 113, 4917–4925. 10.1021/ja00013a030.

[ref54] Gilli P.; Gilli G. The Nature of the Hydrogen Bond: Outline of a Comprehensive Hydrogen Bond Theory; Oxford University Press: New York, NY, USA, 2009.

[ref55] Bertolasi V.; Ferretti V.; Gilli P.; Gilli G.; Issa Y. M.; Sherif O. E. Intramolecular N–H··· O hydrogen bonding assisted by resonance. Part 2. Intercorrelation between structural and spectroscopic parameters for five 1,3-diketone arylhydrazones derived from dibenzoylmethane. J. Chem. Soc., Perkin Trans. 2 1993, 2, 2223–2228. 10.1039/P29930002223.

[ref56] Domagała M.; Simon S.; Palusiak M. Resonance-assisted hydrogen bond—revisiting the original concept in the context of its criticism in the literature. Int. J. Mol. Sci. 2022, 23, 233 10.3390/ijms23010233. PMC8745518 35008659

[ref57] Krygowski T. M.; Bankiewicz B.; Czarnocki Z.; Palusiak M. Quasi-aromaticity - What does it mean?. Tetrahedron 2015, 71, 4895–4908. 10.1016/j.tet.2015.05.074.

[ref58] Palusiak M.; Simon S.; Solà M. Interplay between intramolecular resonance-assisted hydrogen bonding and local aromaticity II 1,3-dihydroxyaryl-2-aldehydes. J. Org. Chem. 2009, 74, 2059–2066. 10.1021/jo802498h. 19195998

[ref59] Palusiak M.; Simon S.; Solà M. The proton transfer reaction in malonaldehyde derivatives: Substituent effects and quasi-aromaticity of the proton bridge. Chem. Phys. 2007, 342, 43–54. 10.1016/j.chemphys.2007.09.016.

[ref60] Palusiak M.; Simon S.; Solà M. Interplay between intramolecular resonance-assisted hydrogen bonding and aromaticity in o-hydroxyaryl aldehydes. J. Org. Chem. 2006, 71, 5241–5248. 10.1021/jo060591x. 16808511

[ref61] Pareras G.; Palusiak M.; Duran M.; Solà M.; Simon S. Tuning the Strength of the Resonance-Assisted Hydrogen Bond in o-Hydroxybenzaldehyde by Substitution in the Aromatic Ring. J. Phys. Chem. A 2018, 122, 2279–2287. 10.1021/acs.jpca.7b12066. 29378123

[ref62] Chuang W. T.; Hsieh C. C.; Lai C.; Lai C.; Shih C.; Chen K. Y.; Hung W. Y.; Hsu Y. H.; Chou P. T. Excited-State Intramolecular Proton Transfer Molecules Bearing o-Hydroxy Analogues of Green Fluorescent Protein Chromophore. J. Org. Chem. 2011, 76, 8189–8202. 10.1021/jo2012384. 21942211

[ref63] Chen K. Y.; Tsai H. Y.; Lin W. C.; Chu H. H.; Weng Y.; Chan C. ESIPT fluorescent dyes with adjustable optical properties: Substituent and conjugation effects. J. Lumin. 2014, 154, 168–177. 10.1016/j.jlumin.2014.04.029.

[ref64] Liu Z. Y.; Hu J. W.; Chen C.; Chen Y. A.; Chen K. Y.; Chou P. T. Correlation among Hydrogen Bond, Excited-State Intramolecular Proton-Transfer Kinetics and Thermodynamics for −OH Type Proton-Donor Molecules. J. Phys. Chem. C 2018, 122, 21833–21840. 10.1021/acs.jpcc.8b07433.

[ref65] Frisch M. J.; Trucks G. W.; Schlegel H. B.; Scuseria G. E.; Robb M. A.; Cheeseman J. R.; Scalmani G.; Barone V.; Mennucci B.; Petersson G. A.; Nakatsuji H.; Caricato M.; Li X.; Hratchian H. P.; Izmaylov A. F.; Bloino J.; Zheng G.; Sonnenberg J. L.; Hada M.; Ehara M.; Toyota K.; Fukuda R.; Hasegawa J.; Ishida M.; Nakajima T.; Honda Y.; Kitao O.; Nakai H.; Vreven T.; Montgomery J. A. Jr.; Peralta J. E.; Ogliaro F.; Bearpark M.; Heyd J. J.; Brothers E.; Kudin K. N.; Staroverov V. N.; Kobayashi R.; Normand J.; Raghavachari K.; Rendell A.; Burant J. C.; Iyengar S. S.; Tomasi J.; Cossi M.; Rega N.; Millam J. M.; Klene M.; Knox J. E.; Cross J. B.; Bakken V.; Adamo C.; Jaramillo J.; Gomperts R.; Stratmann R. E.; Yazyev O.; Austin A. J.; Cammi R.; Pomelli C.; Ochterski J. W.; Martin R. L.; Morokuma K.; Zakrzewski V. G.; Voth G. A.; Salvador P.; Dannenberg J. J.; Dapprich S.; Daniels A. D.; Farkas Ö.; Foresman J. B.; Ortiz J. V.; Cioslowski J.; Fox D. J. Gaussian 09, Revision D.01; Gaussian, Inc.: Wallingford, CT, 2009.

[ref66] Becke A. D. Density-functional thermochemistry V. Systematic optimization of exchange-correlation functionals. J. Chem. Phys. 1997, 107, 8554–8560. 10.1063/1.475007.

[ref67] Karibayev M.; Myrzakhmetov B.; Kalybekkyzy S.; Wang Y.; Mentbayeva A. Binding and Degradation Reaction of Hydroxide Ions with Several Quaternary Ammonium Head Groups of Anion Exchange Membranes Investigated by the DFT Method. Molecules 2022, 27, 2686 10.3390/molecules27092686. 35566033 PMC9104685

[ref68] Shmukler L. E.; Fedorova I. V.; Gruzdev M. S.; Safonova L. P. Triethylamine-Based Salts: Protic Ionic Liquids or Molecular Complexes?. J. Phys. Chem. B 2019, 123, 10794–10806. 10.1021/acs.jpcb.9b08032. 31765153

[ref69] Wang N. X.; Wilson A. K. The behavior of density functionals with respect to basis set I. The correlation consistent basis sets. J. Chem. Phys. 2004, 121, 7632–7646. 10.1063/1.1792071. 15485223

[ref70] Boese A. D. Density functional theory and hydrogen bonds: are we there yet?. ChemPhysChem 2015, 16, 978–985. 10.1002/cphc.201402786. 25688988

[ref71] Kruszewski J.; Krygowski T. M. Definition of aromaticity basing on the harmonic oscillator model. Tetrahedron Lett. 1972, 13, 3839–3842. 10.1016/S0040-4039(01)94175-9.

[ref72] Krygowski T. M.; Cyrański M. K. Structural Aspects of Aromaticity. Chem. Rev. 2001, 101, 1385–1420. 10.1021/cr990326u. 11710226

[ref73] Krygowski T. M.; Szatylowicz H. Aromaticity: what does it mean?. ChemTexts 2015, 1, 12 10.1007/s40828-015-0012-2. 30637186 PMC6313370

[ref74] Safari J.; Gandomi-Ravandi S. Structure, synthesis and application of azines: a historical perspective. RSC Adv. 2014, 4, 46224–46249. 10.1039/C4RA04870A.

[ref75] CrysAlisPRO software system. Oxford Diffraction; Agilent Technologies UK Ltd: Yarnton, England, 2015.

[ref76] Dolomanov O. V.; Bourhis L. J.; Gildea R. J.; Howard J. A. K.; Puschmann H. OLEX2: A Complete Structure Solution, Refinement and Analysis Program. J. Appl. Crystallogr. 2009, 42, 339–341. 10.1107/S0021889808042726.

[ref77] Sheldrick G. M. SHELXT - Integrated space-group and crystal-structure determination. Acta Crystallogr., Sect. A: Found. Adv. 2015, 71, 3–8. 10.1107/S2053273314026370. 25537383 PMC4283466

[ref78] Sheldrick G. M. Crystal structure refinement with SHELXL. Acta Crystallogr., Sect. C: Struct. Chem. 2015, 71, 3–8. 10.1107/S2053229614024218. 25567568 PMC4294323

[ref79] Spek A. L. Structure validation in chemical crystallography. Acta Crystallogr., Sect. D: Biol. Crystallogr. 2009, 65, 148–155. 10.1107/S090744490804362X. 19171970 PMC2631630

[ref80] The Cambridge Crystallographic Data Centre, 12, Union Road, Cambridge CB2 1EZ, UK, http://www.ccdc.cam.ac.uk/conts/ retrieving.html.

[ref81] Hammett L. P. Physical Organic Chemistry; McGraw-Hill: New York, 1940.

[ref82] Grabowski S. J. What Is the Covalency of Hydrogen Bonding?. Chem. Rev. 2011, 111, 2597–2625. 10.1021/cr800346f. 21322583

[ref83] Jabłoński M. A Critical Overview of Current Theoretical Methods of Estimating the Energy of Intramolecular Interactions. Molecules 2020, 25, 5512 10.3390/molecules25235512. 33255559 PMC7728086

[ref84] Mukhopadhyay A.; Xantheas S. S.; Saykally R. J. The water dimer II: Theoretical investigations. Chem. Phys. Lett. 2018, 700, 163–175. 10.1016/j.cplett.2018.03.057.

[ref85] Etter M. C. Encoding and decoding hydrogen-bond patterns of organic compounds. Acc. Chem. Res. 1990, 23, 120–126. 10.1021/ar00172a005.

