# Supplementary material for: Effect of a Substituent on the Properties of Salicylaldehyde Hydrazone Derivatives
Source: J Org Chem. 2023 Feb 3;88(4):2132–9. doi: 10.1021/acs.joc.2c02547 (PMC9942203; doi:10.1021/acs.joc.2c02547)
Supplement: Supplementary file 1 — jo2c02547_si_001.pdf [file jo2c02547_si_001.pdf]

Supporting Information (SI)

The effect of a substituent on the properties of salicylaldehyde hydrazone derivatives

Marta Hoelm[1], Justyna Adamczyk[2], Kinga Wzgarda-Raj[1], Marcin Palusiak[1]*

[1]Department of Physical Chemistry, Faculty of Chemistry, University of Lodz, Pomorska 163/165, Lodz, 90-236,Poland

[2] Department of Organic and Applied Chemistry, Faculty of Chemistry, University of Lodz, Tamka 12, Lodz, 91-403 ,Poland

** Corresponding author e-mail: marcin.palusiak@chemia.uni.lodz.pl*

Table of Contents:

Figure S1. ^{1}H-NMR for 2-hydroxy-5-(*N*-phenylanilino)benzaldehyde……………………………………S4

Figure S2. ^{1}H-NMR and ^{13}C-NMR for compound (**I**)…………………………………………………...S5

Figure S3. ^{1}H-NMR and ^{13}C-NMR for compound (**II**)……………………………………………………S6

Figure S4. IR for compound (**I**)…………………………………………………………………………S7

Figure S5. IR for compound (**II**)………………………………………………………………………...S7

Table S1. Details of the crystal structure determination of (**I**) and (**II**).……………………………………S8

Figure S6. Molecular structure of (**I**) and (**II**) with atom labeling scheme.….……….……………………..S9

Table S2. Geometric parameters for crystal structure (**I**)……...………………………………..………....S9

Table S3. Geometric parameters for crystal structure (**II**).……………………..……..…………………..S10

Figure S7. Scheme of hydrogen bonds presented in the crystal structures (**I**) and (**II**)………….……….S11

Table S4. Geometric parameters of selected hydrogen bonds for crystal structures (**I**) and (**II**)………….....S12

Figure S8. View of the chain of molecules by (**II**) – (**II**) intermolecular interactions and view of the unit cell showing the herringbone arrangement of molecules………………...………………………………………S12

Table S5. Total energies as well as the energy differences ($\Delta\_E_{c/o}$) calculated between closed and open tautomeric forms of the analysed molecules obtained from the B3LYP-GD3/aug-cc-pVTZ calculations……………………………..……………………………………………………………...S13

Figure S9. The correlation between energy difference ($\Delta\_E_{c/o}$) and the Hammett's constant (R) for the investigated systems………………………………………………………………………………………S14

Table S6. Graphical representation and coordinates of the analysed molecules obtained from the B3LYP-GD3/aug-cc-pVTZ calculations……………………...…………………..................................S15

Table S7. Values of HOMA index calculated for benzene and the quasi-ring……………………...…..…S26

References…………………………………………………………..………………………………...S26

1. Materials, Synthesis and Spectroscopic methods

All chemicals and solvents for the synthesis of salicylaldehyde derivatives were purchased from Merck Company and used without additional purification. The 5-trifluoromethoxybenzaldeyhe for synthesis compound 172 was commercially available.

Melting point was determined by MPH-H2 apparatus.

The ^{1}H, ^{13}C -NMR spectra were recorded on a Bruker instrument at 600 MHz and 150 MHz, respectively. Chemical shifts (δ) are given in ppm and coupling constants *J* in Hz. Data are reported as s = singlet, d = doublet, t = triplet, q = quartet, m = multiplet, br. s = broad singlet. Assignments of signals in ^{13}C-NMR spectra were made on the basic of HMQC experiments. Column chromatography was performed using Merck 60 silica gel.

The IR Spectra were recorded on Agilent Technologies Cary 630 FTIR in solid films; absorption in cm^{-1}.

1.1. Synthesis of 2-hydroxy-5-(*N*-phenylanilino)benzaldehyde

Pd(OAc)2 (0.038 g, 0.17 mmol, 3.00% mol), tri-*tert*-butylphosphine (0.104 g, 0.51 mmol, 9% mol), and *ca.* 5 ml of anhydrous toluene under argon were added to a round bottom flask. The mixture was stirred until the palladium acetate dissolved and the solution turned yellow. Then 5-bromosalicylaldehyde (1.21 g, 6 mmol, 1.05 eq), diphenylamine (0.966 g, 5.71 mmol, 1.00 eq) and sodium *tert*-butoxide (0.824 g, 8.57 mmol, 1.5 eq) were added. Upon addition of another portion of anhydrous toluene (25 ml) it was heated to 110 °C and left overnight. Then the reaction was cooled to room temperature and quenched with water. The resulting suspension was extracted with diethyl ether and dried over magnesium sulphate. The organic layer was evaporated and the crude mixture was purified by flash chromatography using hexane/dichloromethane (2:1) to afford the title compound as a yellow oil.

Yellow oil (30%); 0.495 g

^{1}H-NMR (600 MHz, $CDCl_3$): δ= 10.88 (s, 1H, O**H**), 9.77 (s, 1H, C**H**O), 7.40-7.27 (m, 6H, C**H**$_{AR}$), 7.08-6.96 (m, 7H, C**H**$_{AR}$)

1.2. Typical procedure of the synthesis of salicylaldehyde hydrazones

An aqueous hydrazine (1eq) was added into a solution of salicylaldehyde (1eq) in ethanol (10-15 ml) and the mixture was stirred in room temperature for 4-24 h. The resulting precipitate was filtered off and the solvent was evaporated to obtain additional portion of product. Hydrazones were purified by crystallization from methanol.

1.3. (1E)-2-hydroxy-5-(*N*-phenylanilino)benzaldehyde hydrazone (I)

Pale yellow solid (27%); 0.513 g, m.p. 151.2-151.7°C.

IR (solid film): 3391*m* (NH), 3034*w br.* (OH), 1580*w*, 1483*s* (N=N), 1379*m*, 1259*s*, 752*s*, 693*s*.

^{1}H-NMR (600 MHz, $CDCl_3$): δ= 10.94 (s, 1H, O**H**), 7.75 (s, 1H, N=C**H**),7.25-7.22 (m, 4H, C**H**$_{AR}$), 7.06-6.91 (m, 9H, C**H**$_{AR}$), 5.45 (s, 2H, N**H**$_2$).

^{13}**C-NMR** (600 MHz, $CDCl_3$): δ= 154.4 (1**C**q), 148.1 (2 **C**q_{AR}), 146.2 (N=**C**H), 139.3 (1**C**q_{AR}), 129.1, 128.5, 126.9, 122.8, 121.9 (12 **C**H_{AR}), 119.1 (1**C**q), 117.57 (1**C**H_{AR}).

Anal. Calcd. for $C_{19}H_{17}N_3O$ (%): C, 75.23; H, 5.65; N, 13.85; O, 5.27 found: C, 75.26; H, 5.77; N, 13.78; O, 5.19.

1.4. (1E)-2-hydroxy-5-(trifluoromethoxy)benzaldehyde hydrazone (II)

Orange solid (91%); 0.093 g, m.p. 81.7-82.2 ℃

IR (solid film): 3392*m* (NH), 3298*w*, 3071*w br.* (OH), 2918*w*, 1602*w*, 1490*m* (N=N), 1293*m*, 1200s, 1133*s*, 924*m*, 786*m*, 700*m*.

^{1}**H-NMR** (600 MHz, $CDCl_3$): δ= 11.09 (s, 1H, O**H**), 7.85 (s, 1H, N=C**H**),7.10-6.95 (m, 3H, C**H**$_{AR}$), 5.57 (s, 2H, N**H**$_2$).

^{13}**C-NMR** (600 MHz, $CDCl_3$): δ= 156.2 (1**C**q), 144.8 (N=**C**H), 141.3 (1**C**q_{AR}), 122.9, 121.5 (2 **C**H_{AR}), 120.6 (1**C**q (**C**F_3), *J*=254 Hz), 118.8 (1**C**q), 117.5 (1**C**H_{AR}).

Anal. Calcd. for $C_8H_7F_3N_2O$ (%): C, 43.65; H, 3.20; N, 12.72; found: C, 43.60; H, 3.27; N, 12.92.

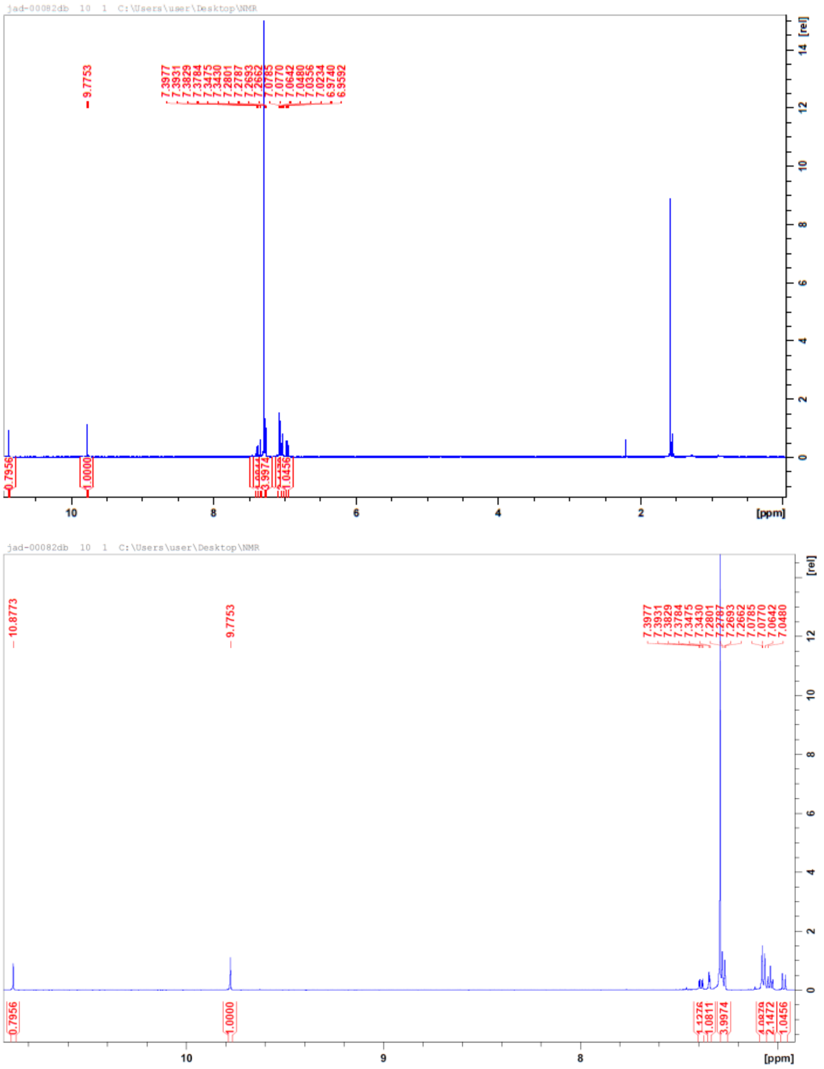

Figure S1. ^{1}H-NMR for 2-hydroxy-5-(*N*-phenylanilino)benzaldehyde.

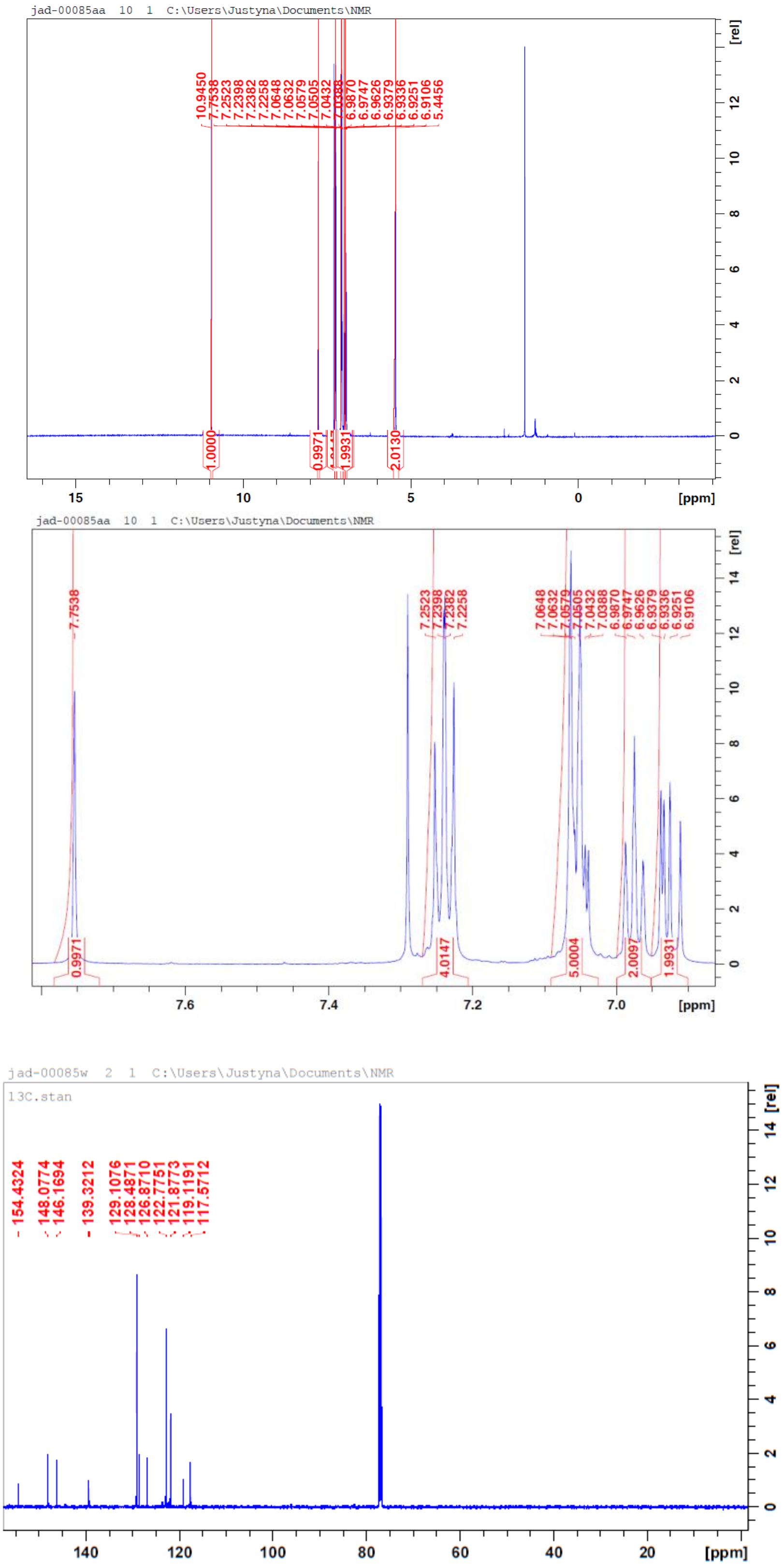

Figure S2. ^{1}H-NMR and ^{13}C-NMR for compound (**I**).

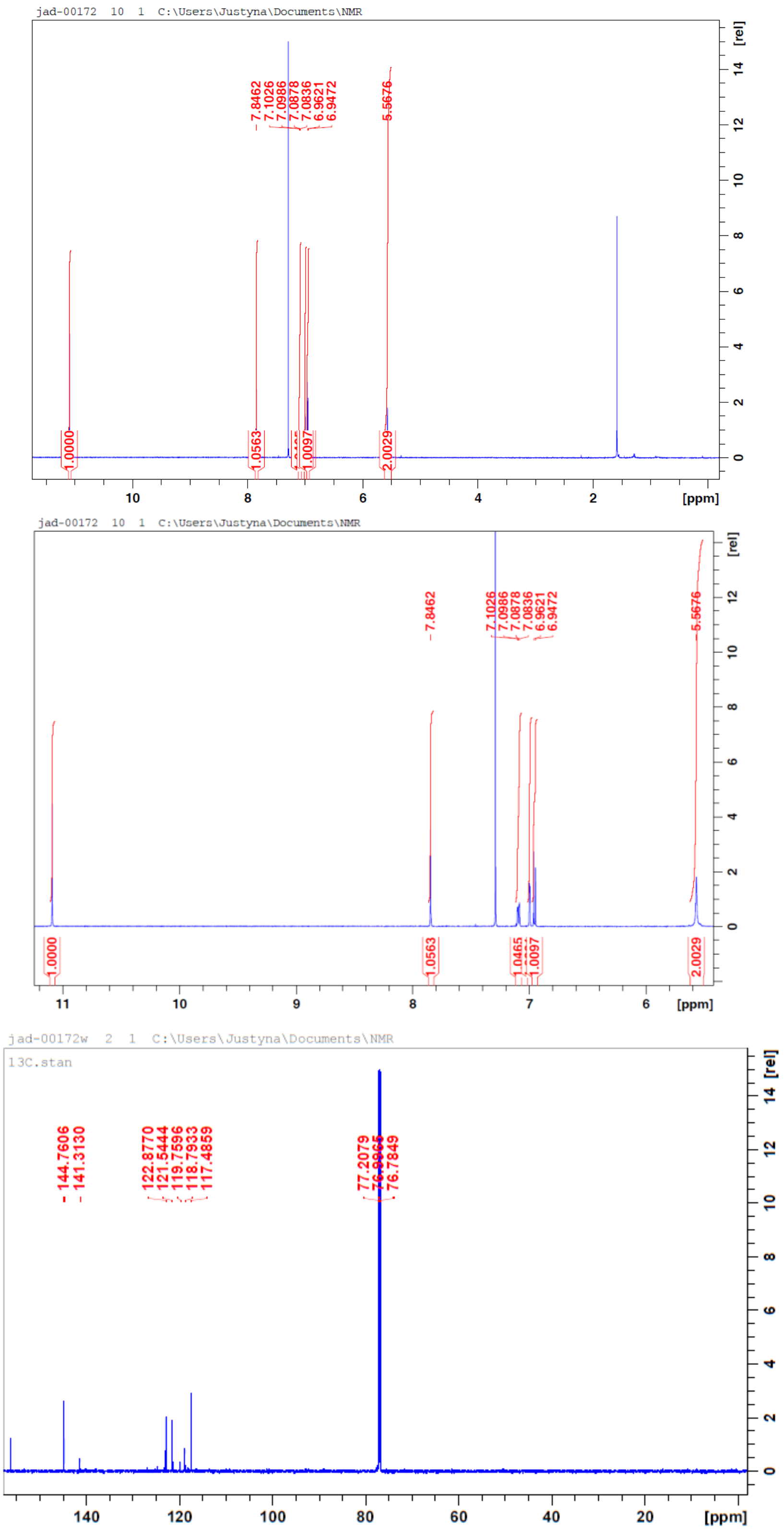

Figure S3. ^{1}H-NMR and ^{13}C-NMR for compound (**II**).

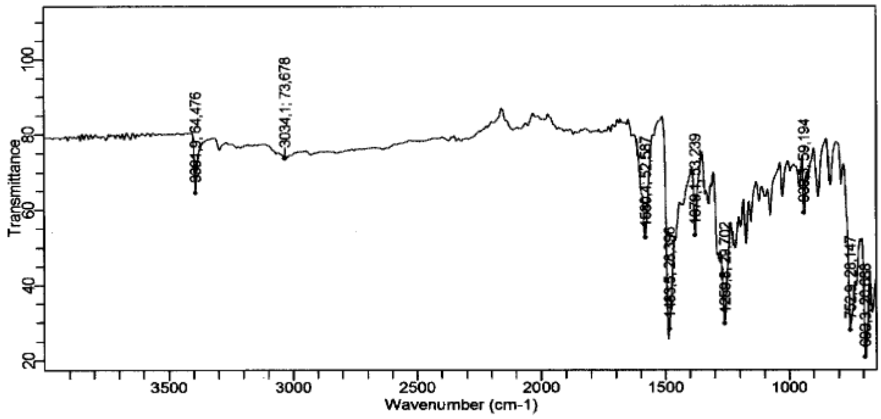

Figure S4. IR for compound (**I**).

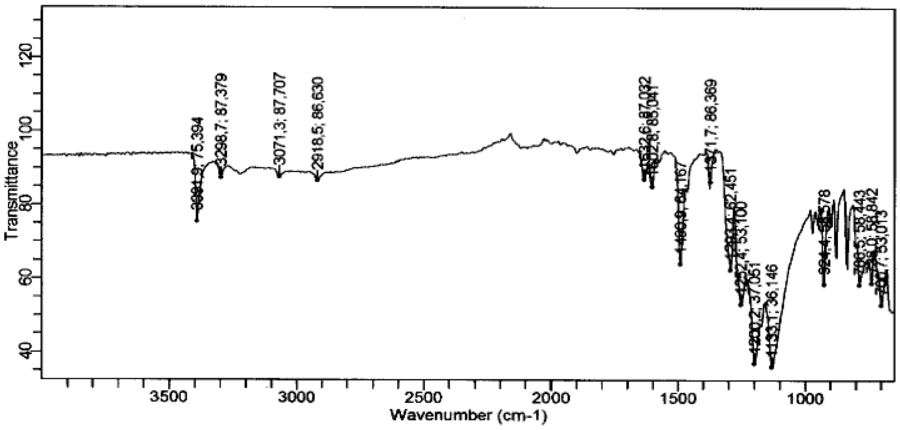

Figure S5. IR for compound (**II**).

2. Crystallographic research

Compound **(I)** crystallizes in centrosymmetric monoclinic space groups $P2_1/c$ and (**II**) – in *C2/c* with one molecule in the asymmetric unit. Molecular diagrams with an atom labeling scheme are presented in **Figure S6**. The details of the crystallographic data of structures (**I**) and (**II**) are given in **Table S1.**

Table S1. Details of the crystal structure determination of **(I)** and **(II)**.

Crystal structure	(I)	(II)
**Empirical formula**	$C_{19}H_{17}N_3O$	$C_8H_7F_3N_2O_2$
**Formula weight**	303.35	220.16
**Temperature/K**	100(2)	100(2)
**Crystal system**	monoclinic	monoclinic
**Space group**	$P2_1/c$	*C2/c*
**a/Å**	10.0110(2)	27.3736(12)
**b/Å**	14.2315(2)	4.5651(2)
**c/Å**	11.3369(2)	13.9894(5)
**α/°**	90	90
**β/°**	104.822(2)	96.243(4)
**γ/°**	90	90
**Volume/Å^3**	1561.44(5)	1737.79(13)
**Z**	4	8
**ρ_{calc} / g/cm^3**	1.290	1.683
**μ/mm^1**	0.651	0.163
**F(000)**	640.0	896.0
**Crystal size/mm^3**	0.169 × 0.131 × 0.052	0.295 × 0.072 × 0.018
**Radiation**	Cu Kα (λ = 1.54184)	Mo Kα (λ = 0.71073)
**2Θ range for data collection/°**	9.138 to 136.994	5.86 to 61.852
**Index ranges**	$-11 \leq h \leq 12$	$-33 \leq h \leq 33$
	$-17 \leq k \leq 17$	$-5 \leq k \leq 5$
	$-13 \leq l \leq 11$	$-17 \leq l \leq 17$
**Reflections collected**	14601	19772
**Independent reflections**	2859	1705
	R_{int} = 0.0270; R_{sigma} = 0.0194	R_{int} = 0.0497; R_{sigma} = 0.0215
**Data/restraints/parameters**	2859/0/218	1705/0/149
**Goodness-of-fit on F^2**	1.088	1.072
**Final R indexes [I>=2σ (I)]**	R_1 = 0.0318, wR_2 = 0.0830	R_1 = 0.0311, wR_2 = 0.0841
**Final R indexes [all data]**	R_1 = 0.0380, wR_2 = 0.0845	R_1 = 0.0364, wR_2 = 0.0869
**Largest diff. peak/hole / e Å^{-3}**	0.22/-0.21	0.22/-0.25
**CCDC number**	2214343	2214344

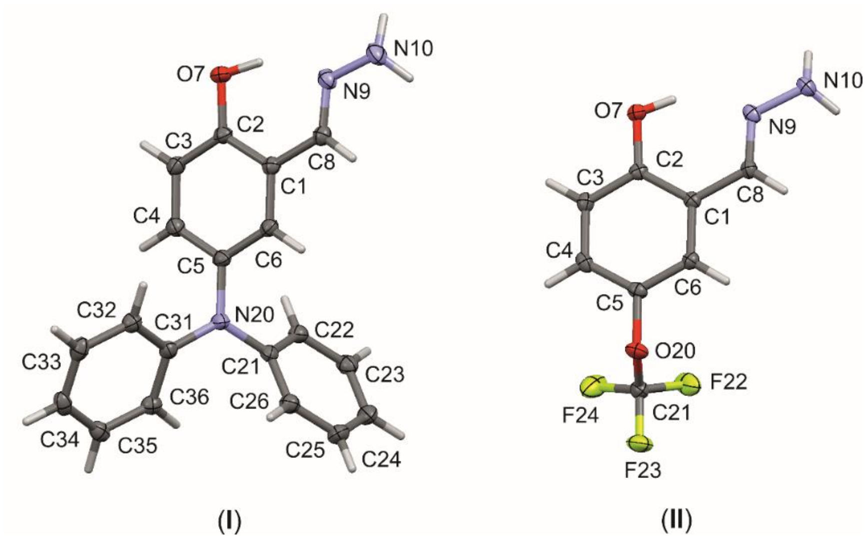

Figure S6. Molecular structure of (**I**) and (**II**) with atom labeling scheme. Displacement ellipsoids are drawn at a 50% probability level.

The C—C bond lengths in the benzene rings in (**I**) and (**II**) are within the expected ranges known for aromatic systems[1]. The N-N bond lengths in the hydrazone functional group (-NNH_2) are comparable within the 3σ criterion (1.386(1)Å and 1.381(1)Å in (**I**) and (**II**), respectively). A similar situation is observed for the C-O bonds in hydroxy- groups (1.365(1)Å in (**I**) and 1.360(1)Å in (**II**)). The bond lengths, angles and torsion angles are given in **Table S2** and **Table S3**.

Table S2. Geometric parameters for crystal structure (**I**).

	**Bond Lengths [Å]**		
O7-C2	1.3650(13)	C32-C31	1.3961(16)
C2-C1	1.4091(15)	C32-C33	1.3856(17)
C2-C3	1.3867(16)	C23-C24	1.3882(17)
C1-C6	1.4017(15)	C23-C22	1.3871(17)
C1-C8	1.4560(15)	C25-C24	1.3885(17)
N20-C5	1.4281(14)	C25-C26	1.3884(16)
N20-C31	1.4185(14)	C31-C36	1.3951(16)
N20-C21	1.4110(14)	C33-C34	1.3863(18)
C5-C4	1.3941(16)	C34-C35	1.3848(17)
C5-C6	1.3866(16)	C36-C35	1.3881(16)
N10-N9	1.3858(14)	C21-C26	1.3987(15)
C4-C3	1.3820(16)	C21-C22	1.4005(16)
N9-C8	1.2833(15)		
	**Bond Angles [°]**		
O7-C2-C1	121.61(10)	C33-C32-C31	120.02(11)
O7-C2-C3	118.49(10)	C22-C23-C24	120.83(11)
C3-C2-C1	119.90(10)	C26-C25-C24	120.94(11)
C2-C1-C8	122.05(10)	C32-C31-N20	120.64(10)
C6-C1-C2	118.46(10)	C32-C31-C36	119.23(10)
C6-C1-C8	119.48(10)	C36-C31-N20	120.12(10)
C31-N20-C5	118.57(9)	C25-C24-C23	118.88(11)

C21-N20-C5	119.19(9)	C32-C33-C34	120.71(11)
C21-N20-C31	122.03(9)	C35-C34-C33	119.37(11)
C4-C5-N20	120.40(10)	C35-C36-C31	120.04(10)
C6-C5-N20	120.42(10)	C34-C35-C36	120.65(11)
C6-C5-C4	119.18(10)	C26-C21-N20	121.36(10)
C3-C4-C5	120.35(11)	C26-C21-C22	118.57(10)
C4-C3-C2	120.72(10)	C22-C21-N20	120.06(10)
C5-C6-C1	121.34(10)	C25-C26-C21	120.32(11)
C8-N9-N10	117.22(10)	C23-C22-C21	120.42(11)
N9-C8-C1	121.39(10)		

Torsion Angles [°]

O7-C2-C1-C6	177.34(10)	C8-C1-C6-C5	-179.55(10)
O7-C2-C1-C8	-1.89(16)	C32-C31-C36-C35	-0.06(17)
O7-C2-C3-C4	-178.39(10)	C32-C33-C34-C35	-0.05(19)
C2-C1-C6-C5	1.19(16)	C31-N20-C5-C4	127.22(12)
C2-C1-C8-N9	-4.91(16)	C31-N20-C5-C6	-53.04(15)
C1-C2-C4-C3	1.16(17)	C31-N20-C21-C26	-26.69(17)
N20-C5-C4-C3	177.81(10)	C31-N20-C21-C22	154.32(11)
N20-C5-C6-C1	-178.89(10)	C31-C32-C33-C34	0.27(18)
N20-C31-C36-C35	178.72(10)	C31-C36-C35-C34	0.27(18)
N20-C21-C26-C25	-178.17(10)	C24-C23-C22-C21	1.31(17)
N20-C21-C22-C23	177.22(11)	C24-C25-C26-C21	0.64(17)
C5-N20-C31-C32	140.18(11)	C33-C32-C31-N20	-178.98(11)
C5-N20-C31-C36	-38.58(15)	C33-C32-C31-C36	-0.21(17)
C5-N20-C21-C26	147.88(11)	C33-C34-C35-C36	-0.22(19)
C5-N20-C21-C22	-31.11(16)	C21-N20-C5-C4	-47.55(15)
C5-C4-C3-C2	0.94(17)	C21-N20-C5-C6	132.20(11)
N10-N9-C8-C1	178.71(10)	C21-N20-C31-C32	-45.21(16)
C4-C5-C6-C1	0.85(16)	C21-N20-C31-C36	136.03(11)
C3-C2-C1-C6	-2.19(16)	C26-C25-C24-C23	-1.15(17)
C3-C2-C1-C8	178.58(10)	C26-C21-C22-C23	-1.80(17)
C6-C1-C8-N9	175.86(10)	C22-C23-C24-C25	0.18(17)
C6-C5-C4-C3	-1.94(17)	C22-C21-C26-C25	0.84(16)

Table S3. Geometric parameters for crystal structure (**II**).

Bond Lengths [Å]

F23-C21	1.3217(16)	C2-C1	1.4106(18)
F22-C21	1.3263(17)	C2-C3	1.3907(18)
F24-C21	1.3328(17)	C1-C6	1.4018(17)
O7-C2	1.3596(15)	C1-C8	1.4582(17)
O20-C5	1.4207(15)	C6-C5	1.3780(18)
O20-C21	1.3355(17)	C5-C4	1.3803(19)
N9-N10	1.3811(16)	C3-C4	1.3867(19)
N9-C8	1.2830(16)		

Bond Angles [°]

C21-O20-C5	116.10(10)	C6-C5-C4	122.21(11)
C8-N9-N10	118.19(10)	C4-C5-O20	119.36(10)

O7-C2-C1	121.54(10)	C4-C3-C2	120.21(11)
O7-C2-C3	117.76(10)	C5-C4-C3	118.82(11)
C3-C2-C1	120.70(11)	F23-C21-F22	107.60(11)
C2-C1-C8	122.25(10)	F23-C21-F24	107.67(10)
C6-C1-C2	118.40(10)	F23-C21-O20	108.46(11)
C6-C1-C8	119.34(10)	F22-C21-F24	106.78(12)
C5-C6-C1	119.65(11)	F22-C21-O20	112.99(10)
N9-C8-C1	121.19(11)	F24-C21-O20	113.09(11)
C6-C5-O20	118.30(11)		
**Torsion Angles [°]**			
O7-C2-C1-C6	179.72(10)	C6-C1-C8-N9	179.91(11)
O7-C2-C1-C8	-1.49(17)	C6-C5-C4-C3	0.70(18)
O7-C2-C3-C4	-179.80(10)	C8-C1-C6-C5	-178.71(10)
O20-C5-C4-C3	176.58(10)	C5-O20-C21-F23	178.66(9)
N10-N9-C8-C1	176.82(10)	C5-O20-C21-F22	59.45(15)
C2-C1-C6-C5	0.12(16)	C5-O20-C21-F24	-62.00(14)
C2-C1-C8-N9	1.12(17)	C3-C2-C1-C6	0.54(16)
C2-C3-C4-C5	-0.02(17)	C3-C2-C1-C8	179.33(11)
C1-C2-C3-C4	-0.60(17)	C21-O20-C5-C6	-96.45(13)
C1-C6-C5-O20	-176.68(10)	C21-O20-C5-C4	87.51(14)
C1-C6-C5-C4	-0.75(18)		

In both crystal structures (**I**) and (**II**), the presence of hydrazone- and hydroxy- groups allows for the formation of two kinds of hydrogen bonds. First, typical intramolecular O7-H7…N9 hydrogen bonds are formed. However, the same donor groups play a role in intermolecular N10-H10A...O7 hydrogen bond between neighboring molecules. Moreover, in structure (**II**), C3-H3…O7 hydrogen bonds result in the creation of a $R_2^2(8)$ intermolecular cyclic motif[2]. **Figure S7** presents the general scheme of hydrogen bonds between molecules in the asymmetric unit, **Table S4** summarizes the geometric parameters of these interactions.

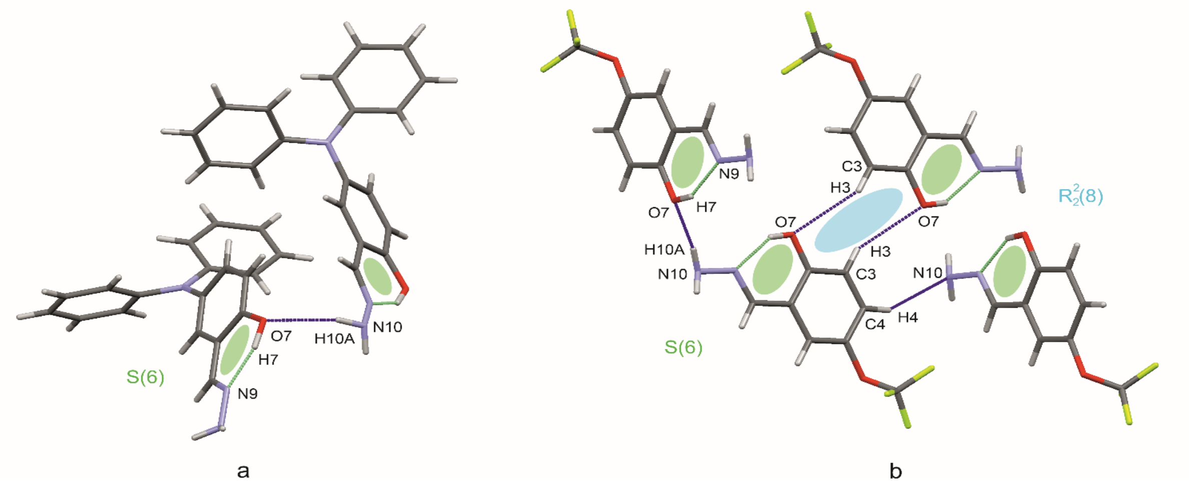

Figure S7. Scheme of hydrogen bonds, presented as blue (intermolecular) and green (intramolecular) dotted lines, in the crystal structures (**I**) - a and (**II**) - b.

Table S4. Geometric parameters of selected hydrogen bonds for crystal structures (**I**) and (**II**) – distances [Å] and angles [°].

**Hydrogen Bond**	**d(D-H)**	**d(H…A)**	**d(D…A)**	**<(D-H…A)**	***Symmetry code***
			**(I)**		
O(7)-H(7)...N(9)	0.840	1.895	2.6340(12)	146.12	-
N(10)-H(10A)...O(7)	0.912(16)	2.369(16)	3.2477(13)	161.8(14)	*x,-y+1/2,z+1/2*
			**(II)**		
O(7)-H(7)...N(9)	0.840	1.889	2.6298(13)	146.39	-
N(10)-H(10A)...O(7)	0.881(16)	2.226(16)	3.1300(12)	167.1(11)	*-x+1,y-1,-z+1/2*
C(3)-H(3)...O(7)	0.95	2.59	3.3425(16)	136.3	*-x+1,-y+2,-z+1*
C(4)-H(4)...N(10)	0.95	2.64	3.5001(18)	150.8	*x,-y+2,z+1/2*

The mentioned above hydrogen bonds are not the only interactions stabilizing the structure (**II**). There are also intermolecular C-O…F interactions (2.895(2)Å), as shown in **Figure S8a**. These interactions of the neighboring -OCF_3 groups are responsible for the formation of infinite chains of molecules extending along [100] crystallographic direction.

Additionally, in structure (**II**), the infinite planar ribbons of molecules extend along the crystallographic [100] direction. In the crystal structure, adjacent ribbons are stacked with corresponding interplanar distances of 2.87(1)Å. A parallel arrangement of the ribbons is additionally stabilized by N-H...O hydrogen bonds. The final supramolecular structure of the ribbons is ordered in a herringbone pattern, as shown in **Figure S8b**.

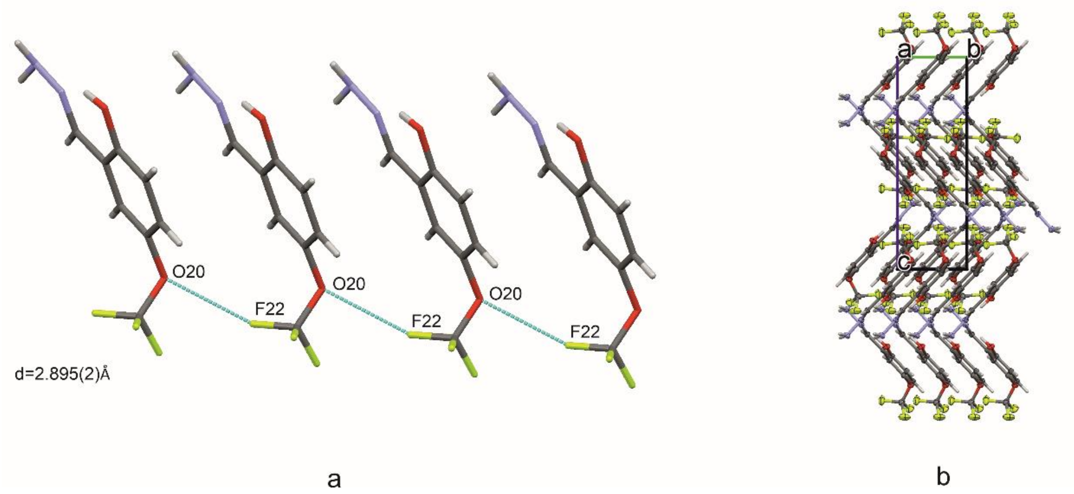

Figure S8. A view of the chain of molecules by (**II**) – (**II**) intermolecular interactions [symmetry code: *x,-1+y,z*] – **a**, and a view of the unit cell showing the herringbone arrangement of molecules - **b**.

3. Quantum-chemical research

Table S5. Total energies as well as the energy differences ($\Delta\_E_{c/o}$) calculated between closed and open tautomeric forms of the analysed molecules obtained from the B3LYP-GD3/aug-cc-pVTZ calculations.

Molecule		Energy of closed structure [hartree]	Energy of open structure [hartree]	$\Delta\_E_{c/o}$ [kcal/mol]
R_1	R_2			
H	CHO	-569.82172	-569.80368	-11.32
	Cl	-916.08109	-916.06386	-10.81
	CN	-548.72630	-548.70864	-11.08
	$N(CH_3)_2$	-590.46796	-590.45120	-10.52
	NH_2	-511.82758	-511.81078	-10.54
	NO	-585.79358	-585.77553	-11.33
	NO_2	-661.03717	-661.01926	-11.24
	OCH_3	-571.01683	-570.99984	-10.66
	OH	-531.70014	-531.68343	-10.48
	$N(C_6H_5)_2$	-974.09922	-974.08204	-10.78
	OCF_3	-868.88155	-868.86425	-10.86
	H	-456.44788	-456.43058	-10.86
CHO	H	-569.81851	-569.80153	-10.66
Cl		-916.08228	-916.06473	-11.02
CN		-548.72440	-548.70764	-10.51
$N(CH_3)_2$		-590.47474	-590.45595	-11.79
NH_2		-511.83403	-511.81515	-11.85
NO		-585.78922	-585.77335	-9.96
NO_2		-661.03373	-661.01777	-10.01
OCH_3		-571.02108	-571.00343	-11.08
OH		-531.70513	-531.68722	-11.24

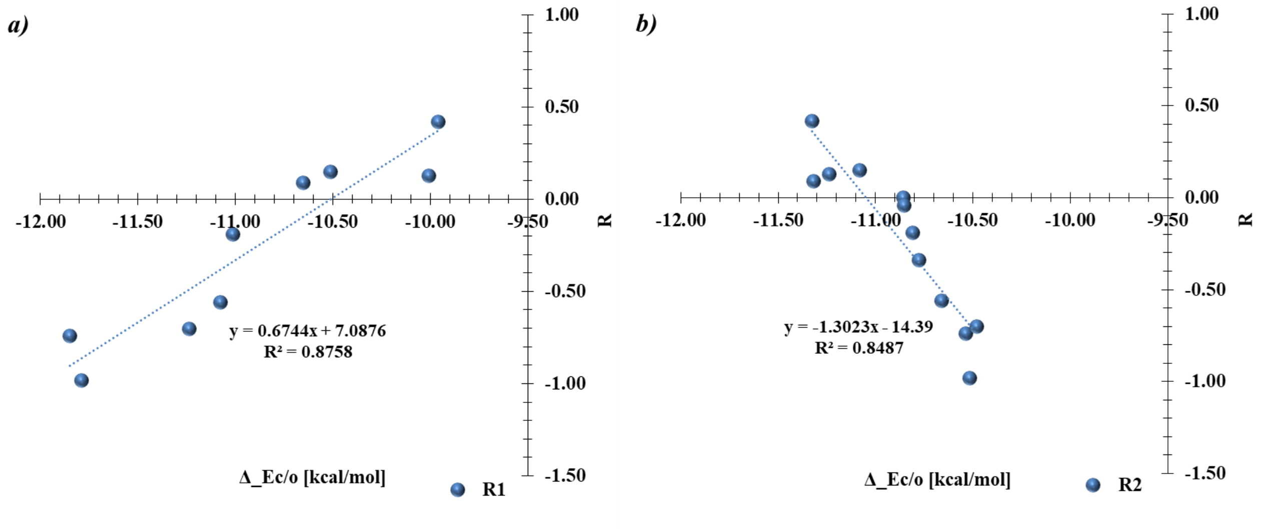

Figure S9. The correlation between the energy difference of closed and open tautomers ($\Delta\_E_{c/o}$) and the Hammett's resonance constant (R) presented for the systems substituted at the R_1 **(a)** and R_2 **(b)** positions, respectively.

Table S6. Graphical representation and coordinates of the analysed molecules obtained from the B3LYP-GD3/aug-cc-pVTZ calculations.

Molecule	Atom	x [Å]	y [Å]	z [Å]
**R₁**=H, **R₂**=CHO				
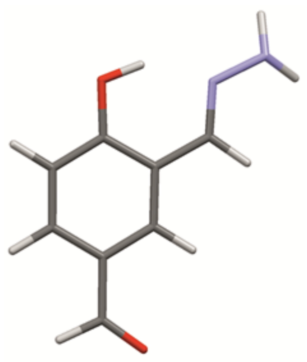	C	0.43866	-0.15583	0.00579
	C	0.51088	1.26428	0.00149
	C	-0.66125	2.02278	-0.00289
	C	-1.89233	1.39667	-0.00472
	C	-1.98649	0.00018	-0.00132
	C	-0.81599	-0.75576	0.00479
	O	1.68337	1.91171	0.00157
	C	1.63167	-0.98725	0.01226
	N	2.81085	-0.48650	-0.00599
	N	3.89845	-1.31324	0.07980
	H	-0.57261	3.09967	-0.00495
	H	-2.79742	1.99167	-0.00869
	C	-3.30146	-0.65382	-0.00389
	H	-0.90304	-1.83506	0.00909
	H	2.40715	1.24208	0.00870
	H	1.48121	-2.06983	0.03914
	H	4.70763	-0.88012	-0.33700
	H	3.74916	-2.25640	-0.26704
	O	-3.48541	-1.85252	-0.00389
	H	-4.16303	0.04512	-0.00628
**R₁**=H, **R₂**=Cl				
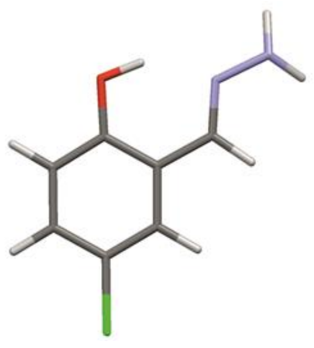	C	-0.54368	-0.18315	-0.00557
	C	-0.55392	1.23074	-0.00155
	C	0.65338	1.92757	0.00319
	C	1.86275	1.25316	0.00522
	C	1.87223	-0.13807	0.00134
	C	0.68895	-0.84922	-0.00466
	O	-1.69828	1.94071	-0.00189
	C	-1.76749	-0.96650	-0.01126
	N	-2.92749	-0.42230	0.00398
	N	-4.04356	-1.21288	-0.08000
	H	0.62206	3.00775	0.00530
	H	2.79454	1.79912	0.00930
	Cl	3.40338	-0.99718	0.00320
	H	0.71252	-1.93023	-0.00925
	H	-2.45150	1.30937	-0.01082
	H	-1.66110	-2.05491	-0.03470
	H	-4.83658	-0.74882	0.33480
	H	-3.92719	-2.15676	0.27792

R$_1$=H, **R$_2$**=CN

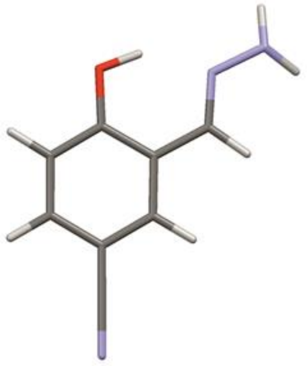

C	0.42342	-0.21372	0.00551
C	0.42912	1.20362	0.00150
C	-0.77934	1.90348	-0.00281
C	-1.98288	1.22906	-0.00466
C	-2.00705	-0.17374	-0.00096
C	-0.80392	-0.87586	0.00489
O	1.56792	1.90971	0.00158
C	1.65220	-0.99092	0.01119
N	2.80674	-0.43541	-0.00566
N	3.93126	-1.20791	0.07870
H	-0.74407	2.98332	-0.00484
H	-2.91308	1.77872	-0.00861
C	-3.24840	-0.87650	-0.00306
H	-0.82189	-1.95760	0.00932
H	2.32418	1.27805	0.00903
H	1.55340	-2.07953	0.03652
H	4.72197	-0.73438	-0.32917
H	3.83087	-2.15753	-0.26766
N	-4.25137	-1.44544	-0.00544

R$_1$=H, **R$_2$**=$N(CH_3)_2$

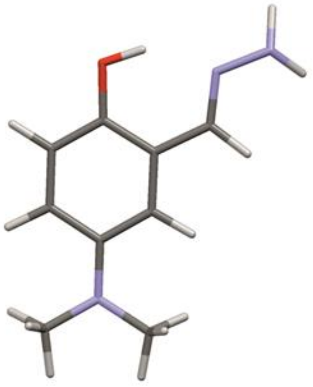

C	0.87600	-0.10350	-0.02802
C	0.97868	1.30065	0.00009
C	-0.19107	2.05199	-0.01216
C	-1.43547	1.44821	-0.05964
C	-1.57273	0.04682	-0.10688
C	-0.39253	-0.70083	-0.07333
O	2.16654	1.94993	0.04732
C	2.04756	-0.96249	-0.00342
N	3.24280	-0.50164	0.02445
N	4.29667	-1.38044	0.12278
H	-0.10819	3.12946	0.02176
H	-2.30654	2.08386	-0.06243
N	-2.83007	-0.56164	-0.20325
H	-0.42969	-1.77958	-0.08494
H	2.87882	1.27671	0.06317
H	1.87029	-2.04289	-0.00553
H	5.12760	-0.96861	-0.27354
H	4.11543	-2.29950	-0.27241
C	-2.91806	-1.98465	0.05997
C	-3.99760	0.22507	0.15078
H	-3.94980	-2.30648	-0.05896
H	-2.31867	-2.54968	-0.65502
H	-2.58505	-2.25537	1.07258
H	-4.88791	-0.38796	0.03325
H	-3.96623	0.59640	1.18483
H	-4.10702	1.08255	-0.51346

R_1=H, **R_2**=NH_2

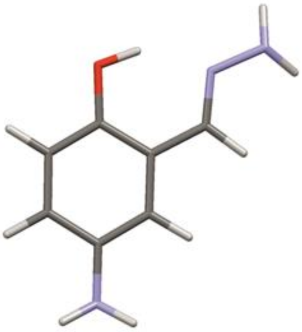

C	0.21619	-0.25994	0.00292
C	0.05529	1.14122	0.00331
C	-1.23168	1.67177	-0.00424
C	-2.34328	0.84672	-0.01257
C	-2.20838	-0.54703	-0.00944
C	-0.92470	-1.07650	-0.00290
O	1.10489	1.99701	0.01097
C	1.52649	-0.88666	0.00860
N	2.61565	-0.21149	0.00191
N	3.81607	-0.87664	0.08297
H	-1.34221	2.74708	-0.00329
H	-3.33237	1.28750	-0.02553
N	-3.34018	-1.37849	-0.07956
H	-0.79225	-2.15255	-0.00751
H	1.92915	1.46632	0.02156
H	1.55256	-1.98094	0.02264
H	4.54784	-0.32263	-0.33484
H	3.80185	-1.81940	-0.29718
H	-3.20236	-2.30535	0.29192
H	-4.18170	-0.96735	0.29314

R_1=H, **R_2**=NO

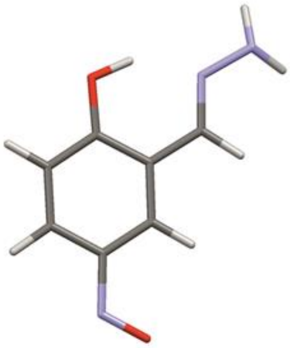

C	0.41706	-0.16676	0.00572
C	0.46656	1.25886	0.00164
C	-0.71552	2.00431	-0.00277
C	-1.93680	1.36336	-0.00487
C	-1.99807	-0.03353	-0.00161
C	-0.82443	-0.78592	0.00462
O	1.62788	1.91916	0.00203
C	1.62515	-0.97704	0.01201
N	2.79418	-0.45307	-0.00633
N	3.89834	-1.25386	0.07879
H	-0.63916	3.08195	-0.00466
H	-2.86328	1.92141	-0.00889
N	-3.30338	-0.59870	-0.00452
H	-0.90380	-1.86519	0.00882
H	2.36224	1.25917	0.00861
H	1.49465	-2.06193	0.03887
H	4.70071	-0.80273	-0.33168
H	3.77231	-2.20196	-0.26315
O	-3.35930	-1.81556	-0.00352

R_1=H, **R_2**=NO_2

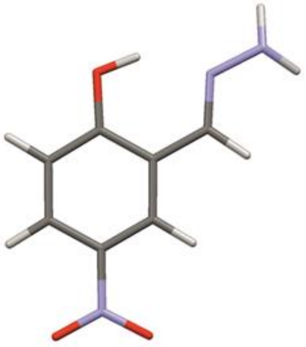

C	0.77518	-0.14985	0.00571
C	0.83893	1.26828	0.00184
C	-0.33939	2.02060	-0.00188
C	-1.57057	1.39865	-0.00354
C	-1.62900	0.00643	-0.00033
C	-0.47672	-0.76064	0.00513
O	2.00482	1.92323	0.00164
C	1.97150	-0.97634	0.01137
N	3.14646	-0.46551	-0.00670
N	4.24104	-1.27861	0.07797
H	-0.25788	3.09772	-0.00366
H	-2.48602	1.96891	-0.00704
N	-2.93139	-0.66105	-0.00243
H	-0.56160	-1.83719	0.00919
H	2.73426	1.25889	0.00821
H	1.82862	-2.05969	0.03781
H	5.04935	-0.83596	-0.32995
H	4.10535	-2.22528	-0.26413
O	-2.94847	-1.88697	-0.00139
O	-3.93441	0.04200	-0.00551

R_1=H, **R_2**=OCH_3

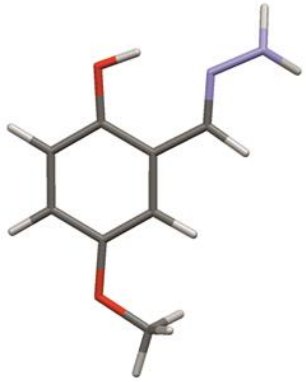

C	-0.49427	-0.11086	-0.00450
C	-0.62032	1.29115	-0.00168
C	0.53214	2.07862	0.00362
C	1.78536	1.50018	0.00665
C	1.92557	0.10816	0.00345
C	0.79127	-0.68555	-0.00235
O	-1.81967	1.91521	-0.00349
C	-1.65078	-0.98888	-0.01012
N	-2.85374	-0.54665	0.00156
N	-3.89661	-1.43809	-0.08218
H	0.41771	3.15338	0.00505
H	2.67769	2.11016	0.01069
O	3.21168	-0.36129	0.00577
H	0.86821	-1.76295	-0.00641
H	-2.52030	1.22827	-0.01413
H	-1.45631	-2.06597	-0.03003
H	-4.72432	-1.04386	0.33787
H	-3.69375	-2.36097	0.29273
C	3.41278	-1.76302	-0.00162
H	4.48909	-1.91404	0.00003
H	2.98192	-2.23553	0.88665
H	2.98592	-2.22552	-0.89700

R$_1$=H, **R$_2$**=OH

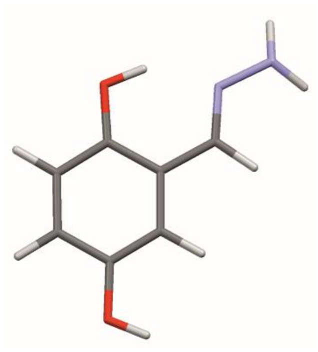

C	0.20833	-0.26661	0.00489
C	0.03243	1.13268	0.00151
C	-1.25895	1.65766	-0.00391
C	-2.36614	0.82809	-0.00643
C	-2.20341	-0.55692	-0.00264
C	-0.92842	-1.09238	0.00315
O	1.07364	1.99573	0.00284
C	1.52389	-0.88136	0.01034
N	2.60565	-0.19426	-0.00162
N	3.81318	-0.84269	0.08146
H	-1.37352	2.73235	-0.00579
H	-3.36613	1.23803	-0.01048
O	-3.34263	-1.32565	-0.00458
H	-0.79474	-2.16868	0.00737
H	1.90343	1.47285	0.01365
H	1.56131	-1.97512	0.03033
H	4.53967	-0.28125	-0.33536
H	3.81278	-1.78880	-0.29013
H	-3.10905	-2.25831	0.00410

R$_1$=H, **R$_2$**=N(C_6H_5)$_2$

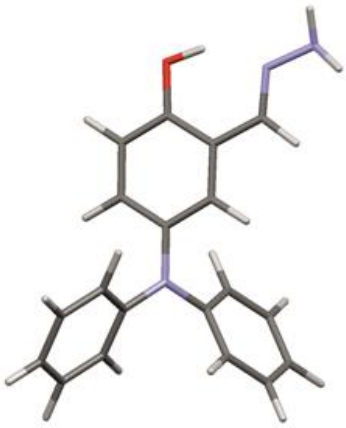

C	2.28886	-3.33789	0.08171
C	1.33266	-3.95274	0.92163
C	0.33717	-3.17314	1.50859
C	0.28379	-1.80895	1.28580
C	1.21410	-1.18462	0.44928
C	2.19791	-1.95996	-0.14665
O	1.34646	-5.27585	1.18208
C	3.35342	-4.09089	-0.55901
N	3.48055	-5.35799	-0.41589
N	4.55514	-5.99421	-0.98556
N	1.15651	0.21826	0.21922
H	-0.38124	-3.65987	2.15292
H	-0.48767	-1.21432	1.75451
H	2.92327	-1.48344	-0.79314
H	2.10577	-5.67400	0.70242
H	4.05946	-3.52541	-1.17455
H	4.33649	-6.96314	-1.15871
H	4.92677	-5.53951	-1.81511
C	2.33061	0.99077	0.36198
C	3.24667	0.70382	1.38030
C	4.41066	1.44810	1.50932
C	2.61130	2.04177	-0.51843
C	3.77095	2.78981	-0.37265
C	4.68113	2.49874	0.63848
C	-0.06710	0.80533	-0.17134
C	-0.94661	0.11779	-1.01493
C	-2.15889	0.68621	-1.38071
C	-2.51464	1.95178	-0.92703

	C	-1.64104	2.64018	-0.09080
	C	-0.43384	2.07423	0.29214
	H	3.04144	-0.10647	2.06507
	H	5.10472	1.21110	2.30477
	H	1.91556	2.26873	-1.31326
	H	3.96938	3.59803	-1.06411
	H	5.58610	3.08037	0.74546
	H	-0.67672	-0.86388	-1.37674
	H	-2.82448	0.13770	-2.03397
	H	-3.45757	2.39350	-1.21764
	H	-1.90616	3.62152	0.27981
	H	0.23215	2.61154	0.95173
**R_1**=H, **R_2**=OCF_3				
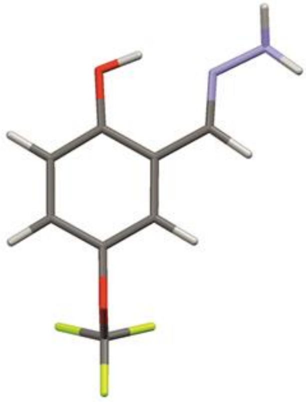	C	1.43357	-0.08743	0.18875
	C	1.64841	1.29072	-0.05107
	C	0.57613	2.18069	0.00973
	C	-0.70014	1.73039	0.30106
	C	-0.90609	0.37715	0.52967
	C	0.13565	-0.52310	0.48123
	O	2.86677	1.78558	-0.33613
	C	2.51241	-1.05965	0.14231
	N	3.72431	-0.72287	-0.10250
	N	4.68509	-1.69208	-0.21051
	O	-2.19156	-0.07513	0.89072
	H	0.76839	3.22775	-0.17431
	H	-1.53457	2.41404	0.35232
	H	-0.05860	-1.56977	0.67001
	H	3.50784	1.04011	-0.34502
	H	2.24737	-2.10574	0.32021
	H	5.59580	-1.31336	-0.00251
	H	4.49784	-2.54313	0.31195
	C	-3.00931	-0.46839	-0.10736
	F	-4.16663	-0.84695	0.43456
	F	-2.51190	-1.50813	-0.80963
	F	-3.25872	0.51385	-0.99651

R$_1$=H, **R$_2$**=H

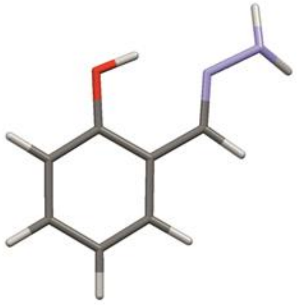

C	-0.07382	-0.45807	0.00418
C	-0.52962	0.88119	0.00167
C	-1.89843	1.14599	-0.00387
C	-2.81407	0.10624	-0.00771
C	-2.38231	-1.21996	-0.00507
C	-1.02493	-1.48775	0.00133
O	0.32067	1.92680	0.00420
C	1.33927	-0.79266	0.01037
N	2.25929	0.09934	-0.00228
N	3.57437	-0.29026	0.08257
H	-2.21846	2.17844	-0.00491
H	-3.87238	0.33019	-0.01217
H	-3.09807	-2.02956	-0.00702
H	-0.67816	-2.51398	0.00495
H	1.23874	1.57609	0.01385
H	1.59592	-1.85633	0.03159
H	4.17056	0.40486	-0.33966
H	3.76437	-1.21768	-0.28761

R$_1$=CHO, **R$_2$**=H

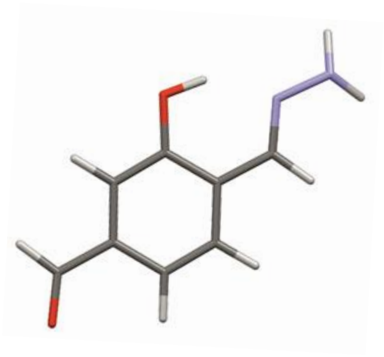

C	0.70848	-0.46594	0.00578
C	0.26021	0.87543	0.00411
C	-1.10652	1.14032	0.00128
C	-2.03163	0.10197	-0.00122
C	-1.59926	-1.22977	0.00052
C	-0.24750	-1.49630	0.00456
O	1.11041	1.91961	0.00481
C	2.11914	-0.80774	0.00917
N	3.03980	0.08531	-0.00574
N	4.34902	-0.29591	0.07426
H	-1.42997	2.17326	0.00089
C	-3.47363	0.42477	-0.00527
H	-2.33363	-2.02170	-0.00050
H	0.09883	-2.52241	0.00743
H	2.02835	1.56915	0.01190
H	2.37352	-1.87118	0.02994
H	4.95221	0.40369	-0.32873
H	4.55226	-1.22854	-0.27339
O	-4.36467	-0.39383	-0.00934
H	-3.70487	1.50917	-0.00456

**R_1**=Cl, **R_2**=H				
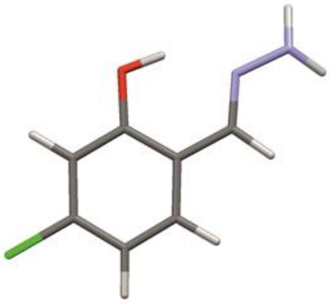	C	-0.78449	0.50415	0.00539
	C	-0.25424	-0.80813	0.00451
	C	1.12582	-1.00344	0.00137
	C	1.96928	0.09278	-0.00165
	C	1.48133	1.39625	-0.00043
	C	0.11060	1.58060	0.00360
	O	-1.04184	-1.89790	0.00638
	C	-2.21357	0.75815	0.00900
	N	-3.07812	-0.18798	-0.00494
	N	-4.41333	0.11787	0.07783
	H	1.51363	-2.01053	0.00140
	Cl	3.70112	-0.16965	-0.00660
	H	2.16062	2.23436	-0.00173
	H	-0.28590	2.58819	0.00601
	H	-1.97932	-1.59837	0.01382
	H	-2.53188	1.80465	0.02968
	H	-4.96665	-0.61451	-0.33945
	H	-4.66310	1.03196	-0.28941
**R_1**=CN, **R_2**=H				
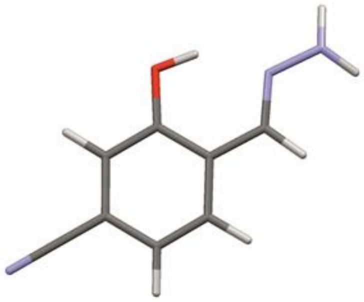	C	-0.65671	0.50127	0.00516
	C	-0.12729	-0.81192	0.00451
	C	1.25084	-1.00328	0.00161
	C	2.11122	0.09259	-0.00150
	C	1.60273	1.39857	-0.00038
	C	0.23480	1.58221	0.00340
	O	-0.91309	-1.90235	0.00621
	C	-2.08601	0.75721	0.00803
	N	-2.94879	-0.19141	-0.00547
	N	-4.27882	0.10439	0.07392
	H	1.63486	-2.01253	0.00173
	C	3.52499	-0.11812	-0.00579
	H	2.27646	2.24188	-0.00177
	H	-0.16641	2.58762	0.00555
	H	-1.85089	-1.60690	0.01320
	H	-2.40470	1.80308	0.02763
	H	-4.83699	-0.63443	-0.32372
	H	-4.54343	1.02135	-0.27372
	N	4.66594	-0.28047	-0.00971

R_1=N(CH$_3$)$_2$, R_2=H				
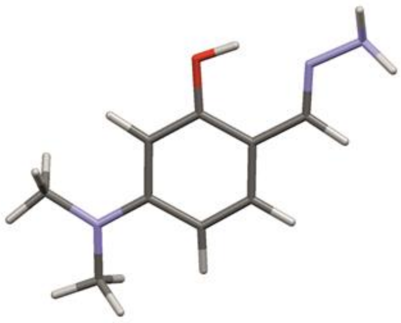	C	1.17097	0.51723	-0.00746
	C	0.61390	-0.78382	-0.01703
	C	-0.76114	-0.96813	-0.03642
	C	-1.64639	0.12275	-0.05724
	C	-1.09216	1.42436	-0.03531
	C	0.27574	1.59354	-0.01429
	O	1.39323	-1.88446	-0.00328
	C	2.59709	0.75167	0.01755
	N	3.45821	-0.19957	0.01369
	N	4.80175	0.11498	0.12027
	N	-3.01090	-0.07056	-0.10339
	H	-1.11288	-1.98684	-0.03476
	H	-1.72530	2.29607	-0.03358
	H	0.67637	2.60016	0.00172
	H	2.33277	-1.59160	0.01796
	H	2.92517	1.79612	0.04524
	H	5.35089	-0.61116	-0.31525
	H	5.04005	1.02315	-0.27112
	C	-3.90485	1.05911	0.06861
	C	-3.55273	-1.40751	0.05175
	H	-4.93264	0.71722	-0.01643
	H	-3.78407	1.54395	1.04475
	H	-3.74325	1.81142	-0.70570
	H	-4.63509	-1.36729	-0.03438
	H	-3.18350	-2.07408	-0.72980
	H	-3.30033	-1.85058	1.02252
**R_1=NH$_2$, R_2=H**				
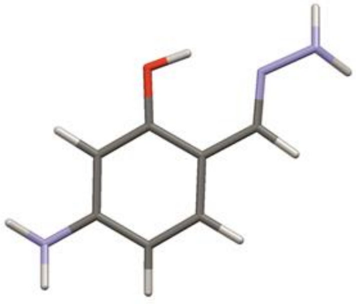	C	-0.38535	0.49046	0.00350
	C	0.12978	-0.82963	-0.00026
	C	1.50094	-1.04894	-0.00631
	C	2.39791	0.01972	-0.00925
	C	1.89881	1.33311	-0.00598
	C	0.53616	1.54571	0.00122
	O	-0.68150	-1.90466	0.00421
	C	-1.80569	0.76522	0.01193
	N	-2.69198	-0.16198	-0.00033
	N	-4.02671	0.18624	0.08945
	H	1.85489	-2.07128	-0.01255
	N	3.76481	-0.21075	-0.06804
	H	2.58332	2.17080	-0.01352
	H	0.16072	2.56184	0.00653
	H	-1.61261	-1.58368	0.01460
	H	-2.10494	1.81824	0.03425
	H	-4.59072	-0.52697	-0.34816
	H	-4.23896	1.10025	-0.30307
	H	4.36008	0.53805	0.24341
	H	4.08216	-1.11843	0.22813

R_1=NO, **R_2**=H

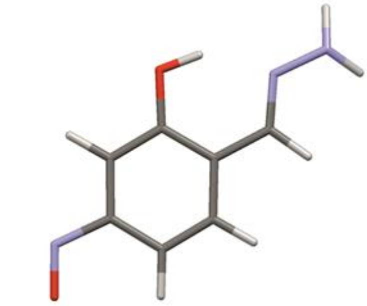

C	0.68125	-0.46037	0.00571
C	0.24233	0.88488	0.00403
C	-1.12250	1.15731	0.00100
C	-2.04060	0.11592	-0.00144
C	-1.62966	-1.22185	0.00054
C	-0.28024	-1.48932	0.00456
O	1.09791	1.92314	0.00483
C	2.08847	-0.81236	0.00874
N	3.01493	0.07584	-0.00547
N	4.31900	-0.31017	0.07241
H	-1.46731	2.18163	0.00039
N	-3.41844	0.52733	-0.00561
H	-2.37055	-2.00674	-0.00037
H	0.06441	-2.51601	0.00743
H	2.01389	1.56865	0.01206
H	2.33646	-1.87706	0.02865
H	4.93034	0.38880	-0.31852
H	4.52292	-1.24561	-0.26644
O	-4.23203	-0.37188	-0.00875

R_1=NO_2, **R_2**=H

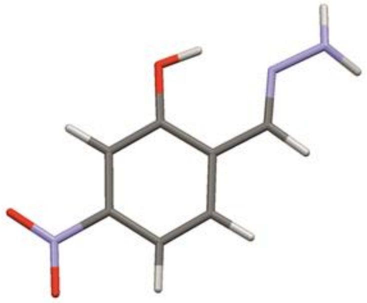

C	-1.04766	0.51392	0.00574
C	-0.51547	-0.79892	0.00513
C	0.86336	-0.98687	0.00250
C	1.69538	0.11807	-0.00034
C	1.20978	1.42094	0.00067
C	-0.15980	1.59850	0.00417
O	-1.29774	-1.89165	0.00661
C	-2.47740	0.76732	0.00810
N	-3.33754	-0.18408	-0.00602
N	-4.66631	0.10565	0.07154
H	1.26850	-1.98585	0.00255
N	3.15604	-0.09881	-0.00441
H	1.89340	2.25361	-0.00062
H	-0.56383	2.60253	0.00617
H	-2.23628	-1.59983	0.01304
H	-2.79900	1.81208	0.02773
H	-5.22476	-0.63642	-0.31922
H	-4.93800	1.02315	-0.26885
O	3.87621	0.89020	-0.00663
O	3.56223	-1.25183	-0.00553

R_1=OCH_3, **R_2**=H

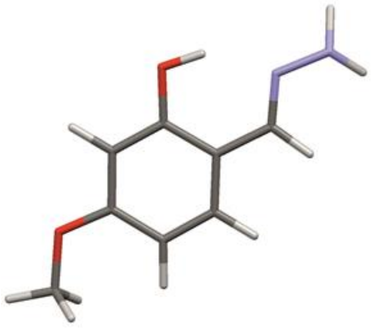

C	-0.78556	-0.45645	-0.00569
C	-0.35062	0.89467	-0.00361
C	1.00381	1.19023	0.00012
C	1.95099	0.16966	0.00219
C	1.54673	-1.16999	-0.00032
C	0.19097	-1.45496	-0.00463
O	-1.22411	1.91959	-0.00509
C	-2.18895	-0.81753	-0.00998
N	-3.12753	0.05540	0.00405
N	-4.43759	-0.36633	-0.08078
H	1.32458	2.22137	0.00095
O	3.24832	0.57683	0.00624
H	2.26287	-1.97543	0.00000
H	-0.12598	-2.49069	-0.00807
H	-2.13414	1.54583	-0.01356
H	-2.42434	-1.88644	-0.03105
H	-5.04371	0.31500	0.35060
H	-4.60078	-1.29321	0.30422
C	4.27361	-0.40594	0.00623
H	5.21171	0.14233	0.00904
H	4.22137	-1.03756	0.89694
H	4.22465	-1.03404	-0.88713

R_1=OH, **R_2**=H

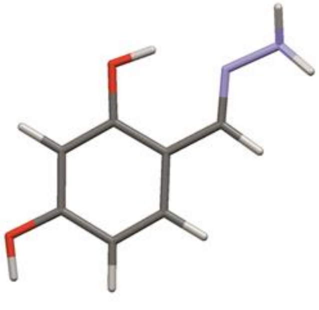

C	0.36807	0.48725	0.00507
C	-0.13522	-0.83871	0.00333
C	-1.50593	-1.06863	-0.00108
C	-2.39001	0.00039	-0.00411
C	-1.91950	1.31646	-0.00193
C	-0.55500	1.53856	0.00311
O	0.68327	-1.90615	0.00571
C	1.78866	0.77372	0.00999
N	2.67921	-0.14817	-0.00346
N	4.00991	0.20192	0.08223
H	-1.87726	-2.08254	-0.00168
O	-3.72215	-0.29650	-0.00889
H	-2.61416	2.14647	-0.00308
H	-0.18525	2.55638	0.00623
H	1.61227	-1.58013	0.01441
H	2.08066	1.82849	0.03108
H	4.57885	-0.51180	-0.34736
H	4.22374	1.11827	-0.30298
H	-4.23818	0.51556	-0.00870

Table S7. The values of HOMA index calculated for benzene and the quasi-ring.

Molecule		HOMA	HOMA quasi-ring
R_1	R_2		
H	CHO	0.938	0.361
	Cl	0.959	0.357
	CN	0.944	0.378
	$N(CH_3)_2$	0.949	0.322
	NH_2	0.961	0.322
	NO	0.923	0.355
	NO_2	0.947	0.384
	OCH_3	0.955	0.349
	OH	0.961	0.338
	$N(C_6H_5)_2$	0.959	0.348
	OCF_3	0.955	0.360
	H	0.957	0.355
CHO	H	0.945	0.373
Cl		0.958	0.379
CN		0.947	0.377
$N(CH_3)_2$		0.914	0.403
NH_2		0.939	0.399
NO		0.938	0.387
NO_2		0.953	0.377
OCH_3		0.948	0.370
OH		0.950	0.384

4. References

(1) Allen, F. H.; Kennard, O.; Watson, D. G.; Brammer, L.; Orpen, A. G.; Taylor, R. Tables of bond lengths determined by X-ray and neutron diffraction. Part 1. Bond lengths in organic compounds. *J. Chem. Soc.*, **1987**, *2*, 1–19.

(2) Etter, M. C. Encoding and decoding hydrogen-bond patterns of organic compounds. *Acc. Chem. Res.*, **1990**, *23*, 120–126.
